# Erosion or Explosion: Integrated Single-Cell Transcriptomics Reveals Cellular Heterogeneity in Aortic Aneurysm and Dissection

**DOI:** 10.1007/s10753-026-02523-5

**Published:** 2026-05-12

**Authors:** Heng Wang, Keyi Fan, Yaling Li, Ziyan Wang, Keyang Xu, Maolin Qiao, Taoran Zhao, Chaonan Fan, Guoping Zheng

**Affiliations:** 1 Centre for Transplant and Renal Research, Westmead Institute for Medical Research, The University of Sydney, Sydney, NSW Australia; 2 Faculty of Medicine and Health, The University of Sydney, Camperdown, NSW Australia; 3 Institute of Vascular Diseases, Shanxi Medical University, Taiyuan, Shanxi China; 4 Department of Vascular Surgery, The Second Hospital of Shanxi Medical University, Taiyuan, Shanxi China; 5 Department of Vascular Surgery, Beijing Anzhen Hospital, Capital Medical University, Beijing, China; 6 Department of Endovascular Surgery, The First Affiliated Hospital of Zhengzhou University, Zhengzhou, Henan China; 7 Faculty of Chinese Medicine, State Key Laboratory of Quality Research in Chinese Medicine, Macau University of Science and Technology, Macau, China; 8 Department of Biochemistry and Molecular Biology, Shanxi Key Laboratory of Birth Defect and Cell Regeneration, MOE Key Laboratory of Coal Environmental Pathogenicity and Prevention, Shanxi Medical University, Taiyuan, Shanxi China

**Keywords:** Aortic aneurysm and dissection, Single-cell RNA sequence, Endothelial cells, Vascular smooth muscle cells, Macrophages, Transdifferentiation

## Abstract

Aortic aneurysm (AA) and aortic dissection (AD) are life-threatening vascular diseases characterized by progressive wall degeneration and structural failure. However, the cellular mechanisms underlying their progression and rupture remain poorly understood. We performed integrative single-cell RNA sequencing analysis on four human aortic datasets, including thoracic aortic dissection (TAD), thoracic aortic aneurysm (TAA), abdominal aortic aneurysm (AAA), and ruptured AAA. We identified disease-specific patterns of immune infiltration and vascular structural cell loss. Vascular smooth muscle cells (VSMCs) and endothelial cells (ECs) were reduced in AA but increased in AD. In ruptured AAA, structural cells were nearly absent, with immune cells predominating. In TAD, VSMCs and ECs exhibited marked phenotypic plasticity, transdifferentiating into multiple cell subtypes. Notably, we identified macrophage-like VSMCs and ECs in AD mouse models. Cell-cell communication analysis revealed that macrophages communicated with VSMCs and ECs via the CXCL, IL1B, and SPP1 signaling axes. Importantly, REL, a member of the NF-κB transcription factor family, was identified as a convergent node integrating these inflammatory signals. This study delineates disease-specific transdifferentiation trajectories of ECs and VSMCs during AA and AD progression. Inflammatory macrophages regulate this phenotypic switching via CXCL, IL1B, and SPP1 signaling, activating REL-driven transcriptional programs that compromise vascular integrity. These findings uncover fundamental differences in cellular heterogeneity and injury mechanisms between AA and AD, offering potential therapeutic targets for preventing disease progression and rupture.

**Graphical Abstract:**

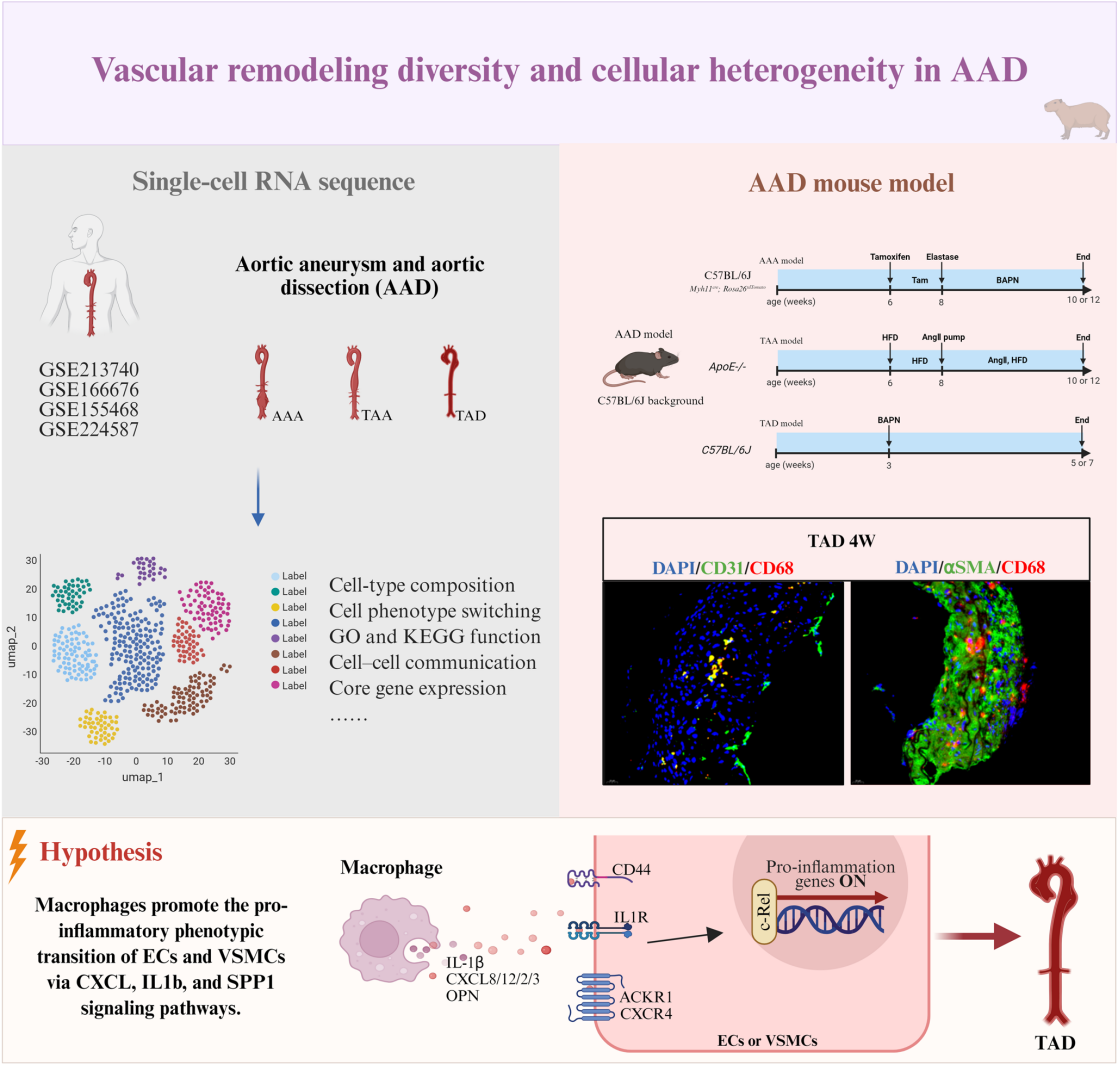

**Supplementary Information:**

The online version contains supplementary material available at 10.1007/s10753-026-02523-5.

## Introduction

Aortic aneurysm (AA) and aortic dissection (AD) are major vascular emergencies associated with high mortality, posing a significant global public health burden [1, 2]. AA is characterized by irreversible, localized or diffuse dilation of the aortic wall, primarily including abdominal aortic aneurysm (AAA) and thoracic aortic aneurysm (TAA) [3, 4]. In contrast, AD is defined by pathological separation of the aortic layers due to intimal and medial tearing, with thoracic aortic dissection (TAD) being the most common subtype [5]. These two diseases share common risk factors, including male sex, aging, smoking, hypertension, atherosclerosis, and genetic predisposition [6–8]. However, there is currently no effective pharmacological therapy, and the mainstay treatments remain open surgery and endovascular stent grafting [8, 9]. Therefore, elucidating the molecular mechanisms underlying AD and AA is crucial for identifying potential therapeutic targets. 

Traditionally, extracellular matrix (ECM) remodeling, inflammatory responses, and dysfunction of vascular smooth muscle cells (VSMCs) have been regarded as major contributors to the initiation and progression of both AA and AD [10–12]. However, during AA progression, the ECM undergoes continuous degradation and remodeling, characterized by the loss of elastic fibers and disorganized accumulation of collagen, which increases vascular stiffness and predisposes the aortic wall to rupture [13–15]. In recent years, integrative multi-omics approaches, combining single-cell transcriptomics and metabolomics, have enabled a comprehensive characterization of cellular heterogeneity and dynamic gene expression changes within aortic tissues [10, 16–19]. During AA progression, the proportion of VSMCs declines, their contractile phenotype weakens, and they can transdifferentiate into multiple VSMC subpopulations. Notably, the emergence of inflammatory VSMC phenotypes has been closely associated with aneurysm rupture [10, 17]. In the context of inflammation, Lyve1⁺ tissue-resident macrophages, Cd74^high^ antigen-presenting macrophages, and Il1rn⁺Trem1⁺ pro-inflammatory macrophages have been identified as key drivers of inflammatory progression and rupture in both AA and AD [20]. Another study revealed that interferon-inducible monocytes/macrophages play a pivotal role in AAA development and rupture by activating pyroptosis and inflammation-related signaling pathways through IRF7 [21]. Additionally, neutrophils contribute to the development of AA and AD by releasing neutrophil extracellular traps (NETs), which are also implicated in thrombosis and aortic rupture. Circulating NETs components such as citrullinated histone H3 in plasma have emerged as potential biomarkers for risk stratification and prognostic assessment in patients with AD [22–25].

However, many current studies tend to classify AA and AD collectively as aortic aneurysmal disease (AAD) when investigating their pathogenesis, thereby overlooking the distinct cellular heterogeneity and injury mechanisms between AA and AD, as well as between TAA and AAA. Moreover, during AD progression, the aortic wall exhibits an avalanche-like failure pattern, representing an acute structural collapse distinct from the chronic development of AA [14]. In addition, endothelial cells (ECs) play a critical role in modulating immune cell infiltration [26, 27].

In this study, we identified the phenomenon of EC-to-macrophage-like and VSMC-to-macrophage-like transdifferentiation in TAD through the establishment of multiple murine models. Single-cell RNA sequencing (scRNA-seq) analysis on human TAA, AAA, ruptured AAA (AAA-rupture), and TAD samples to comprehensively characterize cellular heterogeneity and functional differences. Subcluster analysis of ECs and VSMCs in TAD revealed a unique landscape of cellular transdifferentiation. Taken together, our study provides a panoramic view of the cellular composition of human AAD and uncovers the cellular heterogeneity between AA and AD.

## Materials and Methods

### Human Single-Cell Datasets and Standard Analysis

Human scRNA-seq datasets were obtained from the Gene Expression Omnibus (GEO) database, including GSE213740 (6 TAD samples and 3 controls), GSE166676 (4 AAA samples), GSE155468 (8 TAA samples), and GSE224587 (5 ruptured AAA samples). Downstream analyses were conducted using the Seurat package (v4.3.0) in R (v4.2.0) [28]. For each dataset, a gene was retained if it was expressed (non-zero counts) in at least three cells, and a cell was retained if it expressed at least 200 genes. Low-quality cells were filtered out based on commonly used quality control metrics, including the number of detected genes, total UMI counts, and the proportion of mitochondrial gene expression. Gene expression matrices were log-normalized and scaled. Highly variable genes (HVGs) were identified, and dimensionality reduction was performed using principal component analysis (PCA). Clustering was carried out using a shared nearest neighbor (SNN) modularity optimization algorithm. Uniform manifold approximation and projection (UMAP) was then applied using the top principal components to visualize cell clusters in two dimensions.

After optimizing the resolution parameter, cell clusters were manually annotated based on established biological knowledge and canonical marker gene expression [16, 17, 29]. The major cell subtypes and their corresponding marker genes were as follows: ECs: VWF, AQP1, CDH5, CD34; VSMCs: MYH11, PLN, CNN1, ACTA2; Fibroblasts (Fib): DCN, LUM, CFH, FN1; Monocytes/macrophages (Mac): CD14, CD68, C1QA, ITGAM; T cells: CD3D, CD3E, CD3G, IL7R; B cells: CD79A, CD79B, LY9, CD19; Neutrophils (Neu): S100A8, S100A9, FCGR3B; Dendritic cells (DCs): CLEC4C, ITGAX, CLEC9A; Mast cells (MCs): TPSAB1, TPSB2, CPA3, FCER1A; Natural killer (NK) cells: KLRC1, XCL2, XCL1, CMC1.

### Secondary Clustering and Annotation of VSMCs and ECs

Cells initially annotated as VSMCs or ECs from the TAA and TAD datasets were extracted for secondary clustering analysis. The same downstream processing pipeline was applied as described above, including normalization, dimensionality reduction, clustering, and UMAP visualization. Subclusters were then annotated based on the expression of canonical marker genes indicative of distinct phenotypic states. These subtype classifications reflect diverse phenotypic plasticity and potential transdifferentiation pathways observed in vascular remodeling during aortic disease progression. VSMC and EC subpopulations were annotated according to established biological background and published research [10, 30, 31].

VSMC subpopulations were annotated as follows: Contractile VSMCs (VSMCs): MYH11, PLN, ACTA2; Adipocyte-like VSMCs (VSMCs_A): CD36, PPARG, LPL; Endothelial-like VSMCs (VSMCs_E): VWF, PECAM1, CDH5; Fibroblast-like VSMCs (VSMCs_F): DCN, FBLN1, FBLN2; Macrophage-like VSMCs (VSMCs_Ma): LYZ, CD14, ITGAM; Mesenchymal stem cell-like VSMCs (VSMCs_Me): LBH, RERGL, CLMN; Osteogenic VSMCs (VSMCs_O): TNFRSF11A, SNX10, MKI67; Proliferative VSMCs (VSMCs_P): PBK, TACC3, CEP55; T cell-like VSMCs (VSMCs_T): CD3D, CD3E, IL7R.

EC subpopulations were annotated according to transdifferentiation features using lineage-specific markers: Adipocyte-like ECs (ECs_A); Osteogenic ECs (ECs_O); Fibroblast-like ECs (ECs_F); Smooth muscle-like ECs (ECs_S); Lymphatic-like ECs (ECs_L): PROX1, CCL21, FLT4; Macrophage-like ECs (ECs_M); T cell-like ECs (ECs_T).

These subtype annotations reflect the phenotypic plasticity and potential transdifferentiation trajectories of vascular cells during aortic disease progression.

### Functional Enrichment Analysis

Functional enrichment analysis was performed using the clusterProfiler (v3.14.3) and ReactomePA (v1.50.0) packages in R [16]. Differentially expressed genes were analyzed for enrichment in Kyoto Encyclopedia of Genes and Genomes (KEGG) pathways, Reactome pathways, and Gene Ontology (GO) categories, including biological processes (BP), cellular components (CC), and molecular functions (MF). For comparisons across multiple gene sets (e.g., among different cell clusters or subtypes), the compareCluster() function was employed to perform parallel enrichment analysis. Terms with a P-value < 0.05 were considered significantly enriched. Enrichment results and pathways were visualized using dot plots.

### Pseudotime Trajectory Analysis

To infer the dynamic transitions of cellular states, pseudotime trajectory analysis was performed using Scorpius and Monocle 2 [32]. HVGs were selected as input features for trajectory inference. In Scorpius, dimensionality reduction was conducted via multidimensional scaling, and cells were ordered along a linear trajectory fitted through the reduced-dimensional space. In parallel, Monocle 2 was employed to reconstruct branched trajectories using the DDRTree algorithm, allowing identification of potential bifurcation events and lineage relationships. Cells were ordered along the pseudotime axis based on transcriptional similarity, and the root state was defined using known marker gene expression. Genes exhibiting dynamic expression patterns along the pseudotime trajectory were identified and visualized to elucidate stage-specific regulatory programs.

### Cell–Cell Communication Analysis

Cell–cell communication analysis was performed using the CellChat R package (v1.6.1), which infers intercellular signaling networks based on known ligand–receptor interactions [33]. Standardized gene expression matrices and annotated cell types were used as input. The built-in human ligand–receptor interaction database was utilized, and a permutation-based statistical framework was applied to calculate communication probabilities between different cell types. Significant signaling pathways and intercellular interactions were identified using default parameters. The communication networks were visualized using circle plots, heatmaps, and hierarchical clustering of signaling pathways. Comparative analyses were also conducted across different conditions or cell clusters to uncover context-specific communication programs.

### Transcription Factor Activity Analysis

Transcription factor (TF) activity analysis was performed using the DoRothEA R package (v1.18.0), which infers TF activity based on the expression of their target genes (regulons). Standardized gene expression matrices were used as input. The analysis was restricted to high-confidence TF–target interactions (confidence levels A–C) curated in the DoRothEA human regulon database. TF activity scores were computed using the VIPER algorithm, which evaluates the enrichment of regulon targets within the expression profile. The resulting TF activity matrix was used for visualization and comparative analysis across different cell types or experimental conditions to identify differentially active transcription factors.

### Mouse Models of AAD

C57BL/6J, ApoE^−/−^, Myh11^CreERT2/+^, and Rosa26^LSL−tdTomato+/+^ mice were obtained from Shanghai Nannan Model Biotechnology Co., LTD. The breeding strategy for Myh11^CreERT2/+^; Rosa26^tdTomato/+^ mice followed our previous study [34]. All animals were housed in specific pathogen-free facilities under controlled environmental conditions (temperature: 20–22 °C; humidity: 55%; 12-hour light/dark cycle) and provided with sterile food and water. All animal procedures were approved by the Ethics Committee of the Second Hospital of Shanxi Medical University (Approval No. 2024005). All compounds were purchased from MedChemExpress LLC.

Two models were used to induce AA in mice (Supplementary Fig. 1A): A Model [25]: Male ApoE^−/−^ mice aged 8–12 weeks were subcutaneously implanted with osmotic mini-pumps for continuous infusion of angiotensin II (AngII, 1000 ng/kg/min) for 28 days. Mice were fed a high-fat diet (HFD) throughout the modeling period to enhance vascular stress and promote aneurysm formation. E Model [17]: Male C57BL/6J or Myh11^CreERT2/+^; Rosa26^tdTomato/+^ mice (8–12 weeks old) were subjected to periaortic incubation with porcine pancreatic elastase (PPE). Under sterile conditions and appropriate anesthesia, a small gelatin sponge soaked in PPE solution was applied directly to the adventitia of the exposed infrarenal abdominal aorta for 20 min, followed by thorough rinsing with saline. To further enhance extracellular matrix degradation and aneurysm progression, β-aminopropionitrile (BAPN, 0.2% w/v) was administered in the drinking water for 4 weeks.

TAD model (Supplementary Fig. 1B) [35]: Three-week-old male C57BL/6J mice received BAPN (0.6% w/v) in their drinking water ad libitum for 4 weeks. BAPN is a specific and irreversible inhibitor of lysyl oxidase, which impairs cross-linking of collagen and elastin, leading to progressive degeneration of the aortic wall. This treatment predisposes mice to spontaneous aortic dissection, particularly in the thoracic aorta.

### Histological Staining

Mouse aortas were carefully dissected, flushed with phosphate-buffered saline, and fixed in 4% paraformaldehyde at 4 °C for 24–48 h. Following fixation, tissues were dehydrated through a graded ethanol series, cleared with xylene, and embedded in paraffin. Serial 5 μm sections were cut using a rotary microtome and mounted on glass slides for histological staining. Hematoxylin and eosin (HE) staining was performed to evaluate the overall morphology of the aortic wall. Elastic Van Gieson (EVG) staining was used to assess elastic fiber architecture and to detect fragmentation or loss of elastic lamellae. Masson’s trichrome staining was employed to visualize collagen deposition and to assess fibrotic remodeling. All stained sections were scanned using the VS200 slide scanning system (Olympus) to acquire high-resolution whole-slide images.

### Immunohistochemical Staining

Paraffin-embedded aortic sections (5 μm) were deparaffinized, rehydrated, and subjected to antigen retrieval using citrate buffer (pH 6.0). After blocking, the sections were incubated overnight at 4 °C with a primary antibody against α-smooth muscle actin (α-SMA, 1:200; Abcam, ab5694). Detection was performed using DAB (3,3’-diaminobenzidine) chromogen, followed by counterstaining with hematoxylin to visualize nuclei. Stained slides were scanned and analyzed using the Olympus VS200 slide scanning system.

### Immunofluorescence Staining

For dual-target immunofluorescence (IF) staining of paraffin-embedded aortic tissue sections, samples were first deparaffinized, followed by antigen retrieval and blocking with appropriate serum. The first primary antibody was applied and incubated overnight at 4 °C in a humidified chamber. On the second day, sections were incubated with the corresponding FITC-conjugated secondary antibody. After thorough washing, the second primary antibody was added and incubated overnight at 4 °C. On the third day, sections were incubated with a Cy5-conjugated secondary antibody. Nuclei were counterstained with DAPI, and sections were mounted with an antifade mounting medium. For single-target staining, the protocol was correspondingly simplified. Primary antibodies used included: α-SMA (Abcam, ab5694), CD68 (Abcam, ab125212), CD31 (Abcam, ab222783), F4/80 (Servicebio, GB113373), MYH11 (Servicebio, GB15011), LGALS3 (Servicebio, GB151145), IL-1β (Servicebio, GB11113), SM22A (Servicebio, GB11366). Fluorescent images were acquired using the Olympus VS200 slide scanning system.

## Results

### Cellular Landscape of Human Aortic Aneurysm and Dissection

**Fig. 1:**
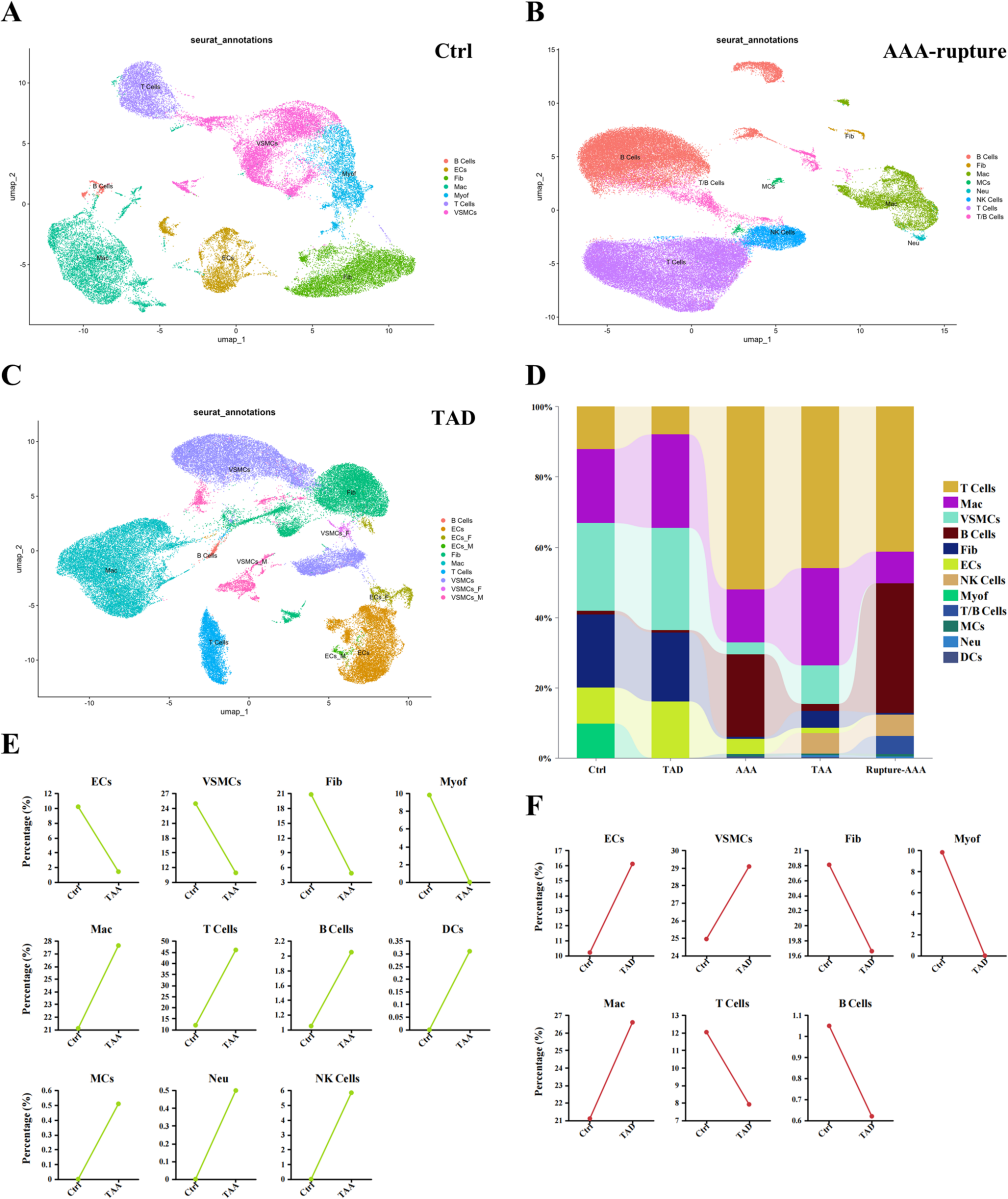
Single-cell landscape of human aortic aneurysm and dissection (AAD) tissues.** (A)** Uniform Manifold Approximation and Projection (UMAP) visualization of single-cell transcriptomic profiles from normal aortic tissue (Ctrl). Each dot represents one cell and is colored according to the annotated cell type identified using Seurat-based clustering and canonical marker genes. Major cell populations include endothelial cells (ECs), vascular smooth muscle cells (VSMCs), fibroblasts (Fib), macrophages (Mac), T cells, and B cells. **(B)** UMAP plot showing cellular clusters from ruptured abdominal aortic aneurysm (AAA) tissue. Distinct immune and vascular cell populations were identified, including B cells, macrophages, neutrophils (Neu), natural killer (NK) cells, fibroblasts, and T/B cells. **(C)** UMAP visualization of single-cell clusters from thoracic aortic dissection (TAD) tissue. Additional subpopulations of vascular structural cells were identified, including endothelial subtypes (ECs_F, ECs_M) and VSMC subsets (VSMCs_F, VSMCs_M), along with immune populations such as macrophages and T cells. **(D)** Stacked bar plot showing the relative proportions of major cell types across different aortic conditions, including Ctrl, TAD, abdominal aortic aneurysm (AAA), thoracic aortic aneurysm (TAA), and ruptured AAA. Each color represents a distinct cell population, and the percentage indicates the fraction of cells belonging to each cell type within the corresponding dataset. **(E)** Quantification of changes in the proportions of vascular structural cells and immune cell populations in TAA compared with control aortic tissue. The y-axis indicates the percentage of cells belonging to each cell type. **(F)** Quantification of changes in the proportions of vascular structural cells and immune cell populations in TAD compared with control aortic tissue

To comprehensively characterize cellular heterogeneity during the progression of aortic aneurysm and dissection, we analyzed four publicly available human scRNA-seq datasets from the GEO database. Specifically, GSE213740 comprised 3 thoracic aortic control samples (37,945 cells) and 6 TAD samples (66,983 cells); GSE224587 included 5 AAA-rupture samples (71,719 cells); GSE166676 contained 4 unruptured AAA samples (7,888 cells); and GSE155468 included 8 unruptured TAA samples (39,545 cells). All datasets underwent standardized processing using the Seurat R package, including quality control, dimensionality reduction, clustering, integration, and manual annotation of cell types. In the control group, cells were clustered into: B cells, T cells, ECs, fibroblasts, myofibroblasts, macrophages, and VSMCs **(**Fig. 1A; Supplementary Fig. 2A). In AAA-rupture, clusters included: B cells, T cells, T/B cells, fibroblasts, macrophages, MCs, neutrophils, and NK cells **(**Fig. 1B; Supplementary Fig. 2B). In the TAD group, clustering revealed: B cells, T cells, macrophages, fibroblasts, ECs, VSMCs, as well as ECs_F, ECs_M, VSMCs_F, and macrophage-like VSMCs (VSMCs_M) **(**Fig. 1C; Supplementary Fig. 2C). For AAA-rupture, cell types included: B cells, T cells, macrophages, fibroblasts, ECs, VSMCs, MCs, and DCs (Supplementary Fig. 3A). For TAA, clusters consisted of: B cells, T cells, macrophages, fibroblasts, ECs, VSMCs, MCs, DCs, NK cells, and neutrophils (Supplementary Fig. 3B).

Compared to the control group, TAA tissues showed a reduced proportion of ECs (1.42%), VSMCs (10.89%), and fibroblasts (4.81%) **(**Fig. 1D, E**)**. Similarly, in AAA tissues, these three structural cell types were also markedly decreased, comprising 4.23%, 3.31%, and 0.56%, respectively **(**Fig. 1D**)**. In contrast, ECs and VSMCs displayed an increasing trend in TAD samples **(**Fig. 1F**)**. Immune cells, particularly macrophages, were notably enriched in both TAA and TAD groups. Notably, in the ruptured AAA group, fibroblasts accounted for only 0.57% of all cells, with the remainder predominantly composed of immune cell types **(**Fig. 1D**)**.

### Disease-Specific Gene Regulatory Networks in AAD

To uncover both the shared and disease-specific gene regulatory programs, we performed GO, KEGG, and cell–cell communication analyses on scRNA-seq datasets from AAA, TAA, ruptured AAA, and TAD samples. We first focused on vascular-resident cells, particularly ECs and VSMCs. In AAA, TAA, and TAD tissues, both ECs and VSMCs were consistently enriched for pathways related to phenotypic switching, ECM remodeling, and collagen biosynthesis. BP analysis revealed enrichment in terms such as endothelial cell differentiation, collagen metabolic process, muscle cell differentiation, and extracellular matrix organization (Supplementary Table 1). CC terms included tight junction, collagen-containing extracellular matrix, and protein complex involved in cell adhesion (Supplementary Table 1). MF terms were enriched for extracellular matrix structural constituent, collagen binding, integrin binding, and transmembrane receptor protein tyrosine kinase activity (Supplementary Table 1). Notably, during the initial clustering and annotation of TAD, ECs were further classified into three subtypes: normal ECs, ECs_F, and ECs_M; VSMCs were subdivided into contractile VSMCs, VSMCs_F, and VSMCs_M **(**Fig. 1C**)**. In contrast, such subtype distinctions were not observed in AAA or TAA, suggesting a more complex vascular cell reprogramming in TAD. Importantly, ECs_M and VSMCs_M in TAD exhibited transcriptional profiles reminiscent of pro-inflammatory macrophages, with enrichment in pathways such as leukocyte transendothelial migration, cell adhesion molecules, and endothelial cell migration (Supplementary Fig. 4–7). In comparison, ruptured AAA samples were devoid of ECs and VSMCs, with only fibroblasts identified among the structural cell types.

We next focused on the immune cell compartments across AAA, TAA, TAD, and AAA-rupture samples. In AAA, TAA, and TAD, macrophages exhibited transcriptional signatures associated with cytokine release, immune cell activation, and phagocytosis, indicating their central role in vascular inflammation and remodeling. In AAA-rupture, both macrophages and neutrophils showed strong enrichment for pathways related to NETs formation, implicating NETs in the pathogenesis of AA rupture (Supplementary Fig. 6). Additionally, compared to TAD, macrophages in TAA displayed significantly higher activity in promoting collagen deposition and extracellular matrix accumulation, suggesting a more fibrotic immune phenotype in TAA (Supplementary Table 1).

We further explored cell–cell communication using ligand–receptor interaction modeling. In AAA, macrophages primarily interacted with ECs via the NAMPT–(ITGA5 + ITGB1) signaling axis. For VSMCs, macrophage-derived signals included TGFB1–(TGFBR1 + TGFBR2), NAMPT–(ITGA5 + ITGB1), and LGALS9–CD44 (Supplementary Fig. 8A-C). In TAA, ECs recruited macrophages, neutrophils, and T cells through CXCL12–CXCR4 signaling. Macrophages interacted with ECs via CXCL2/3/8–ACKR1, and with VSMCs primarily through SPP1–CD44/(ITGA8 + ITGB1) signaling (Supplementary Fig. 9A-C). In AAA-rupture, macrophages mainly signaled to other immune cells through the MIF–(CD74 + CXCR2/4/CD44) axis, highlighting a pro-inflammatory communication program (Supplementary Fig. 10A, B). In TAD, the intercellular communication network was more extensive and complex. ECs and VSMCs both engaged other cell types via MIF–(CD74 + CXCR4/CD44) signaling, while macrophages primarily utilized CXCL2/3/8–ACKR1 and SPP1–CD44/(ITGA5/V+ITGB1) axes to mediate interactions with surrounding structural and immune cells (Supplementary Fig. 11A-C).

### Phenotypic Evolution of VSMCs in AA

**Fig. 2:**
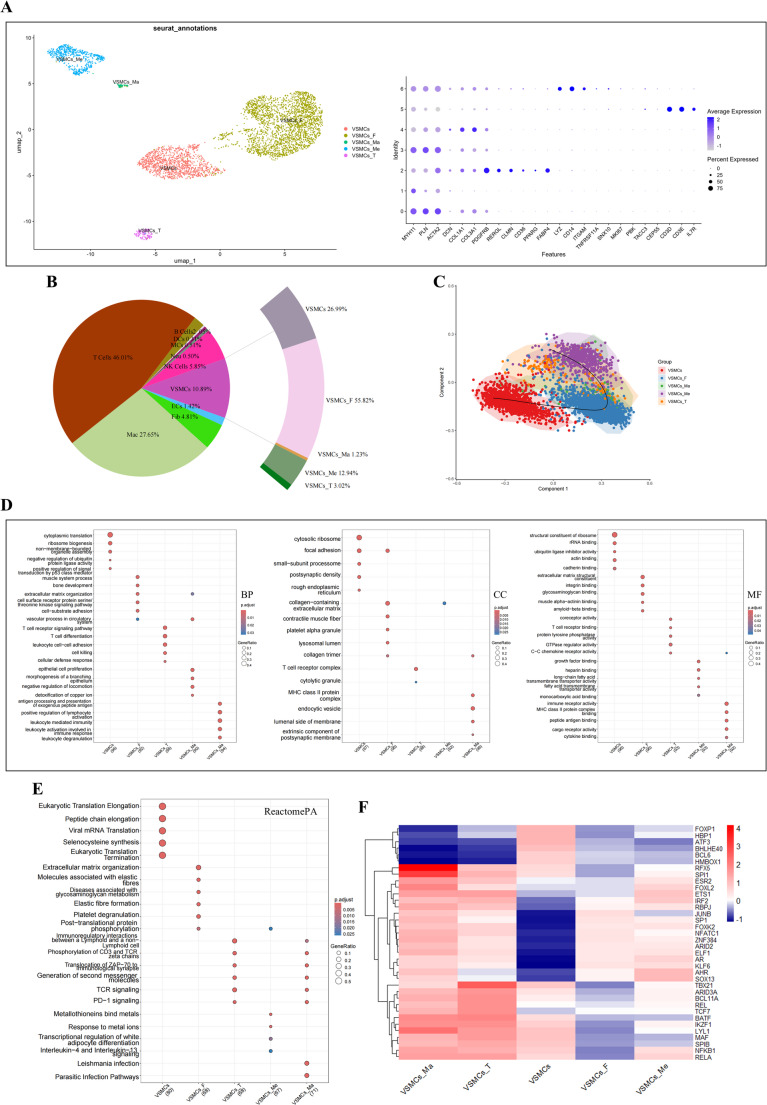
Subclustering and functional characterization of vascular smooth muscle cells (VSMCs) in thoracic aortic aneurysm (TAA) tissue.** (A)** Secondary clustering of VSMCs derived from TAA tissue visualized using Uniform Manifold Approximation and Projection (UMAP). Each dot represents an individual cell, colored according to the identified VSMC subpopulation based on Seurat clustering and canonical marker gene expression. The right panel shows a dot plot of representative marker genes used to annotate the VSMC subclusters. Dot size represents the percentage of cells expressing the gene, and color intensity indicates the average expression level. (**B**) Proportional distribution of major cell types in TAA tissue and relative proportions of the identified VSMC subpopulations. The pie chart illustrates the overall cellular composition of the dataset, while the adjacent ring chart highlights the proportions of VSMC subsets within the VSMC compartment. **(C)** Pseudotime trajectory analysis of VSMC subpopulations, illustrating the inferred differentiation trajectory and potential phenotypic transitions among VSMC subsets. Cells are colored according to their annotated subpopulation identity. **(D)** Gene Ontology (GO) enrichment analysis of differentially expressed genes in each VSMC subpopulation. Enriched terms are categorized into Biological Process (BP), Cellular Component (CC), and Molecular Function (MF). Dot size represents the gene ratio, and color indicates the adjusted p-value. **(E)** Reactome pathway enrichment analysis showing significantly enriched signaling pathways across different VSMC subpopulations. Dot size indicates gene ratio, and color represents statistical significance (adjusted p-value). **(F)** Heatmap showing the predicted key transcription factors associated with each VSMC subpopulation. Rows represent transcription factors and columns represent VSMC subclusters. Color intensity indicates relative expression levels

Given the near-complete loss of ECs and VSMCs in ruptured AAA, we focused our analysis on the cellular heterogeneity in unruptured aneurysms. In both AAA and TAA, the proportions of ECs and VSMCs were markedly reduced compared to control tissues, where ECs accounted for 10.21% and VSMCs for 24.95% of total cells **(**Fig. 1D**)**. Due to the limited number of ECs in aneurysmal tissues, effective secondary clustering was not feasible for ECs. We therefore performed secondary clustering on 4,306 VSMCs from TAA samples, which revealed five distinct subpopulations **(**Fig. 2A**)**: contractile VSMCs, VSMCs_F, VSMCs_Ma, VSMCs_Me, VSMCs_T. Among them, contractile VSMCs, which represent the normal phenotype, accounted for only 26.99% of the total population **(**Fig. 2B**)**. Pseudotime trajectory analysis demonstrated that all other VSMC subtypes originated from this contractile VSMC pool, suggesting a dynamic phenotypic transition during disease progression **(**Fig. 2C**)**.

GO enrichment analysis was performed for each VSMC subtype **(**Fig. 2D; Supplementary Table 2). In the BP category, VSMCs_Ma was enriched in terms including antigen processing and presentation of exogenous peptide antigen, positive regulation of lymphocyte activation, leukocyte-mediated immunity, leukocyte activation involved in immune response, and leukocyte degranulation. In the CC category, enriched terms included MHC class II protein complex, endocytic vesicle, lumenal side of membrane, and extrinsic component of postsynaptic membrane. In the MF category, terms such as immune receptor activity, MHC class II protein complex binding, peptide antigen binding, cargo receptor activity, and cytokine binding were significantly enriched. Further pathway enrichment analysis using ReactomePA revealed that immune-inflammatory signaling was markedly activated in VSMC subpopulations **(**Fig. 2E**)**. A summary of key TFs associated with each VSMC subtype is provided in Fig. 2F.

**Fig. 3:**
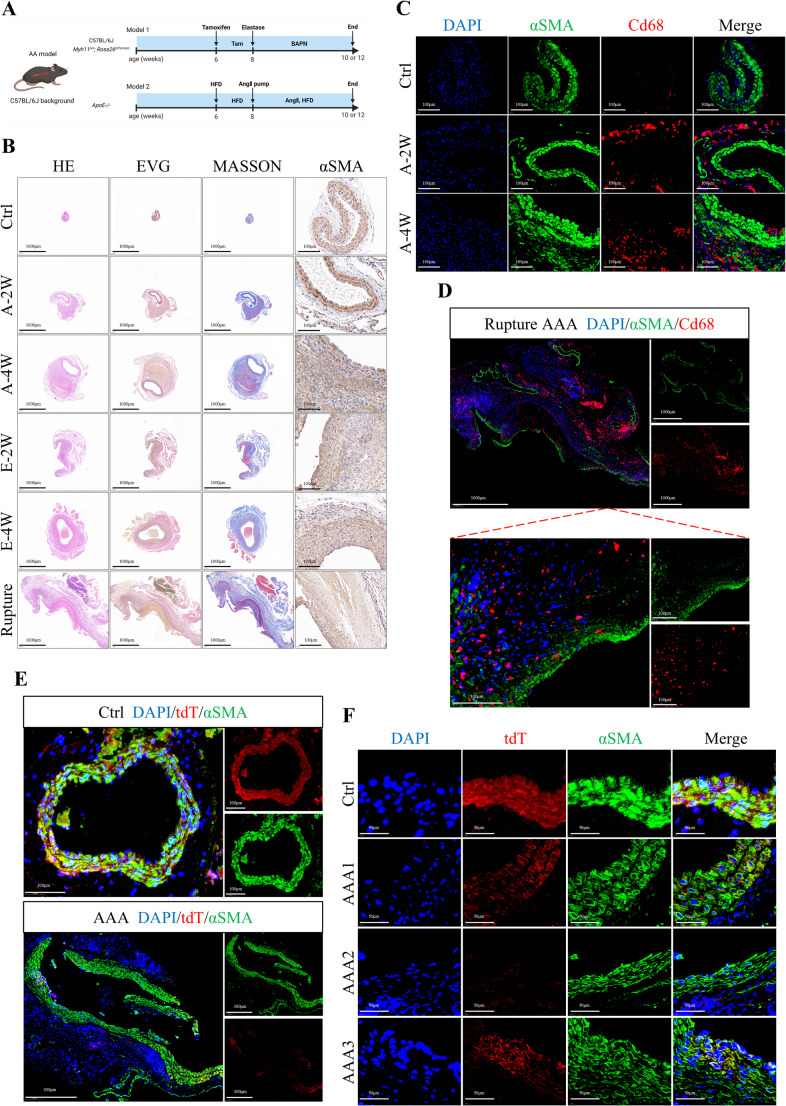
Phenotypic transition of vascular smooth muscle cells (VSMCs) in mouse models of aortic aneurysm (AA).** (A)** Schematic illustration of the experimental design for two mouse models of aortic aneurysm. Model A was established in ApoE⁻/⁻ mice by implantation of an angiotensin II (Ang II)–releasing osmotic pump combined with a high-fat diet (HFD). Model E was induced in C57BL/6J mice by topical application of porcine pancreatic elastase to the abdominal aorta together with administration of β-aminopropionitrile (BAPN). The timeline indicates the induction and progression of aneurysm formation. **(B)** Representative histological staining of aortic sections from both AA mouse models at different time points, including hematoxylin–eosin (HE), elastic van Gieson (EVG), Masson’s trichrome staining, and immunohistochemical staining for α-smooth muscle actin (α-SMA). A-2 W and A-4 W indicate 2 and 4 weeks after Ang II infusion, respectively; E-2 W and E-4 W indicate 2 and 4 weeks after elastase treatment. Scale bars are shown in each panel (*n* = 5 mice per group). **(C)** Immunofluorescence staining of aortic sections from Model A mice showing VSMCs and macrophages. VSMCs were labeled with α-SMA (green), macrophages with CD68 (red), and nuclei with DAPI (blue). Representative images from control (Ctrl), Ang II-treated mice at 2 weeks (A-2 W), and 4 weeks (A-4 W) are shown (*n* = 5). **(D)** Immunofluorescence staining of ruptured abdominal aortic aneurysm (AAA) tissue showing co-localization of α-SMA (green) and CD68 (red) signals. DAPI (blue) marks nuclei. The lower panels show enlarged views of the indicated region, highlighting the presence of α-SMA–positive cells expressing macrophage markers. **(E)** Representative immunofluorescence images of aortic sections from Myh11-CreERT2/+; Rosa26-tdTomato/+ lineage-tracing mice. tdTomato (tdT, red) labels MYH11-expressing VSMCs, α-SMA marks smooth muscle cells (green), and nuclei are stained with DAPI (blue). Images from control and AAA tissues are shown. **(F)** Higher-magnification images of lineage-traced VSMCs in AAA tissues from Myh11-CreERT2/+; Rosa26-tdTomato/+ mice. Co-localization of tdTomato and α-SMA signals indicates VSMC lineage identity during aneurysm progression. Representative images from multiple AAA samples are shown (AAA1–AAA3)

Inflammation is a hallmark of AA progression and rupture. Therefore, we focused on the VSMCs_Ma subpopulation. Two mouse models of AA were utilized **(**Fig. 3A**)**: A model, in which AngII was continuously infused via osmotic minipumps in ApoE^−/−^ mice fed with HFD; E model, in which the abdominal aorta was incubated with PPE, followed by BAPN administration via drinking water. Histological evaluation of murine aortas is shown in Fig. 3B. To assess VSMC–macrophage transdifferentiation, we performed dual immunofluorescence staining using α-SMA (VSMC marker) and CD68 (macrophage marker). At 2 weeks (AA 2 W), there was minimal co-localization of α-SMA and CD68. However, by 4 weeks (AA 4 W), extensive co-expression was observed, indicating a phenotypic shift toward a pro-inflammatory state **(**Fig. 3C**)**. We next conducted immunofluorescence co-staining in aortic tissues from AAA mice using F4/80 with MYH11, LGALS3 with ACTA2, and IL-1β with SM22A. These results indicate that VSMCs express inflammatory mediators and acquire macrophage-like characteristics, supporting a phenotypic transition of VSMCs toward macrophage-like cells (Supplementary Fig. 12). In AAA-rupture, α-SMA expression was markedly diminished, with extensive CD68^+^ macrophage infiltration, highlighting the close association between inflammation and aneurysm rupture **(**Fig. 3D**)**. We further employed Myh11^CreERT2^; Rosa26^tdTomato^ mice for VSMC lineage tracing. During aneurysm progression, fluorescence signals from both tdTomato and α-SMA progressively declined **(**Fig. 3E, F**)**, suggesting that VSMCs underwent transdifferentiation followed by cell loss. These findings indicate that during aneurysm development, VSMCs undergo phenotypic switching toward inflammatory-like states, but ultimately fail to sustain structural identity, leading to VSMC depletion, compromised wall integrity, inflammatory cell infiltration, and vessel rupture.

### Phenotypic Transdifferentiation of ECs in AD

**Fig. 4:**
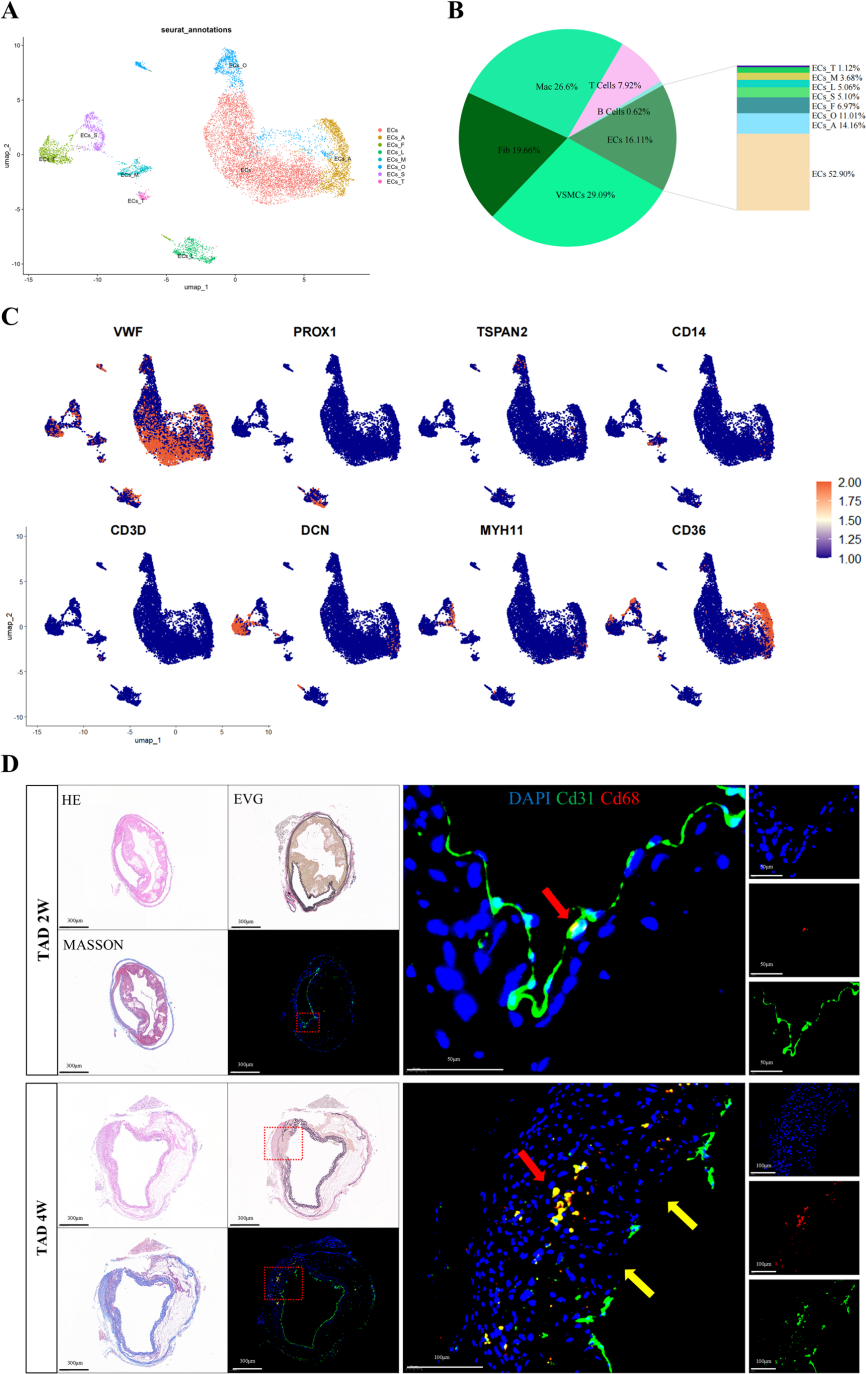
Phenotypic changes of endothelial cells (ECs) in thoracic aortic dissection (TAD).** (A)** Secondary clustering of ECs derived from human TAD tissue visualized using Uniform Manifold Approximation and Projection (UMAP). Each dot represents a single cell and is colored according to the annotated EC subpopulation identified by Seurat-based clustering and canonical marker genes. **(B)** Relative proportions of major cell types and EC subpopulations in the TAD single-cell dataset. The pie chart shows the overall cellular composition of the tissue, while the adjacent stacked bar indicates the distribution of EC subclusters within the EC compartment. **(C)** Feature plots showing the expression patterns of representative marker genes across the UMAP embedding, including endothelial markers (VWF), lymphatic endothelial markers (PROX1), endothelial activation markers (TSPAN2), immune-associated markers (CD14), T-cell markers (CD3D), fibroblast markers (DCN), vascular smooth muscle markers (MYH11), and lipid metabolism–associated markers (CD36). Color intensity represents relative gene expression levels. **(D)** Histopathological and immunofluorescence analysis of aortic tissues from a mouse model of thoracic aortic dissection at 2 and 4 weeks after induction. Representative hematoxylin–eosin (HE), elastic van Gieson (EVG), and Masson’s trichrome staining are shown. Immunofluorescence staining was performed using CD31 (green) to label endothelial cells and CD68 (red) to label macrophages, with nuclei counterstained by DAPI (blue). Enlarged views highlight areas of endothelial disruption and macrophage accumulation within the dissected aortic wall. Arrows indicate representative regions of EC–macrophage interactions (*n* = 5 mice per group)

Given the pronounced transdifferentiation tendencies observed in ECs within TAD tissues, we performed secondary clustering on 10,792 ECs initially annotated from TAD samples **(**Fig. 4A; Supplementary Fig. 13A). Based on differential gene expression and manual curation, ECs were classified into eight distinct subpopulations: Normal ECs, ECs_A, ECs_F, ECs_L, ECs_M, ECs_O, ECs_T, ECs_S. Among them, VWF^+^ ECs accounted for 52.90%, while ECs_M represented 3.68% of the total EC population **(**Fig. 4B, C**)**. We further validated these findings in a TAD mouse model **(**Fig. 4D**)**. At 2 weeks, the vascular endothelium appeared largely intact. Notably, we captured rare ECs co-expressing CD31 and CD68, corresponding to the ECs_M phenotype. By 4 weeks, severe intimal disruption, elastic fiber fragmentation, and collagen deposition were observed, accompanied by ECs_M migration into the lesion site.

Next, we conducted cell–cell communication analysis among the eight EC subtypes (Supplementary Fig. 13B, C). Inter-subtype signaling was extensive, with ECs_M primarily engaging other EC subtypes through the CXCL and SPP1 signaling axes (Supplementary Fig. 13B). Reactome pathway enrichment showed that ECs_M were predominantly associated with immune-related pathways, including: immunoregulatory interactions between a lymphoid and a non-lymphoid cell, neutrophil degranulation, interleukin-10 signaling, FCGR activation (Supplementary Fig. 14). GO enrichment analysis further revealed subtype-specific biological roles. Notably, ECs_M exhibited macrophage-like functional characteristics, such as inflammatory cytokine secretion, immune receptor activation, phagocytosis, and migration, supporting their role in endothelial plasticity and inflammatory transformation during dissection progression (Supplementary Table 3).

### Phenotypic Transdifferentiation of VSMCs in AD

**Fig. 5:**
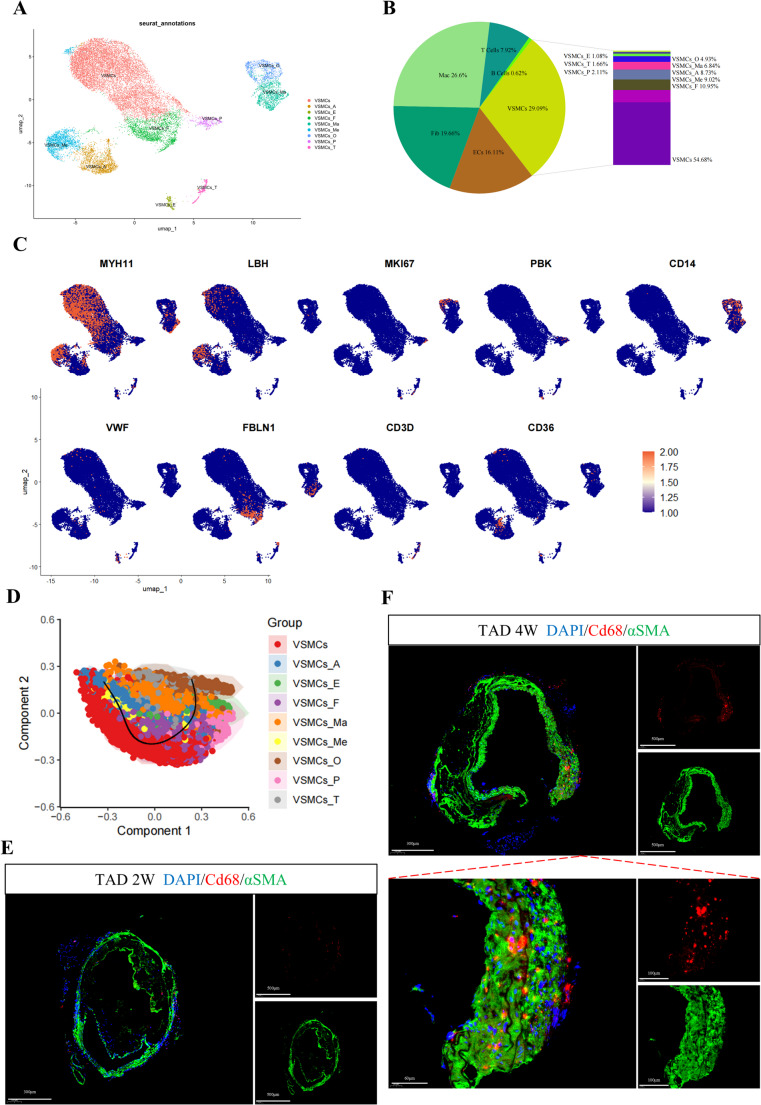
Phenotypic changes of vascular smooth muscle cells (VSMCs) in thoracic aortic dissection (TAD).** (A)** Secondary clustering of VSMCs derived from human TAD tissue visualized using Uniform Manifold Approximation and Projection (UMAP). Each dot represents a single cell and is colored according to the annotated VSMC subpopulation identified by Seurat clustering and canonical marker genes. **(B)** Relative proportions of major cell types and VSMC subpopulations in the TAD single-cell dataset. The pie chart shows the overall cellular composition of the tissue, while the adjacent stacked bar plot illustrates the distribution of VSMC subclusters within the VSMC compartment. **(C)** Feature plots showing the expression patterns of representative marker genes across the UMAP embedding. MYH11 marks contractile VSMCs, LBH indicates synthetic VSMC states, MKI67 and PBK represent proliferative signatures, while CD14, CD36, CD3D, VWF, and FBLN1 indicate immune-related, endothelial, and extracellular matrix–associated features. **(D)** Pseudotime trajectory analysis of VSMC subpopulations illustrating the inferred phenotypic transition and differentiation trajectory among VSMC states. Cells are colored according to their annotated subpopulation identity. **(E**,** F)** Immunofluorescence staining of aortic sections from the TAD mouse model at 2 weeks (TAD 2 W) and 4 weeks (TAD 4 W) after induction. VSMCs were labeled with α-smooth muscle actin (α-SMA, green), macrophages with CD68 (red), and nuclei with DAPI (blue). Enlarged views highlight regions with accumulation of macrophages within the dissected aortic wall and their spatial proximity to α-SMA–positive cells (*n* = 5 mice per group)

To explore phenotypic plasticity of VSMCs in TAD, we performed secondary clustering on 19,489 VSMCs identified from TAD tissues **(**Fig. 5A; Supplementary Fig. 15A). Based on marker gene expression and annotation, VSMCs were classified into nine subtypes: contractile VSMCs, VSMCs_A, VSMCs_E, VSMCs_F, VSMCs_Ma, VSMCs_Me, VSMCs_O, VSMCs_P, VSMCs_T. Among them, contractile VSMCs accounted for 54.68%, while VSMCs_Ma represented 6.84% of the total population **(**Fig. 5B**)**. Pseudotime trajectory analysis revealed that all non-contractile VSMC subtypes originated from the contractile VSMC lineage **(**Fig. 5C, D**)**, indicating a dynamic phenotypic transformation during dissection progression. We further validated these findings in the TAD mouse model. At 2 weeks, a small number of α-SMA^+^CD68^+^ cells (VSMCs_Ma) were observed **(**Fig. 5E**)**. By 4 weeks, VSMCs_Ma was more abundant and localized to areas of aortic wall thickening **(**Fig. 5F**)**, supporting their role in lesion development.

Subsequent cell–cell communication analysis among the nine VSMC subtypes revealed that VSMCs_Ma actively signaled through the CXCL, IL1B, and SPP1 pathways (Supplementary Fig. 15B-D). Similar to ECs_M, VSMCs_Ma showed enrichment in immune-related signaling pathways, including: immunoregulatory interactions between a lymphoid and a non-lymphoid cell, neutrophil degranulation, phosphorylation of CD3 and TCR zeta chains, costimulation by the CD28 family, TCR signaling (Supplementary Fig. 16). GO analysis further highlighted that VSMCs_Ma were primarily involved in immune activation responses (Supplementary Table 3).

### Macrophage-Mediated Regulation of EC and VSMC Phenotypic Switching Drives TAD Progression

**Fig. 6:**
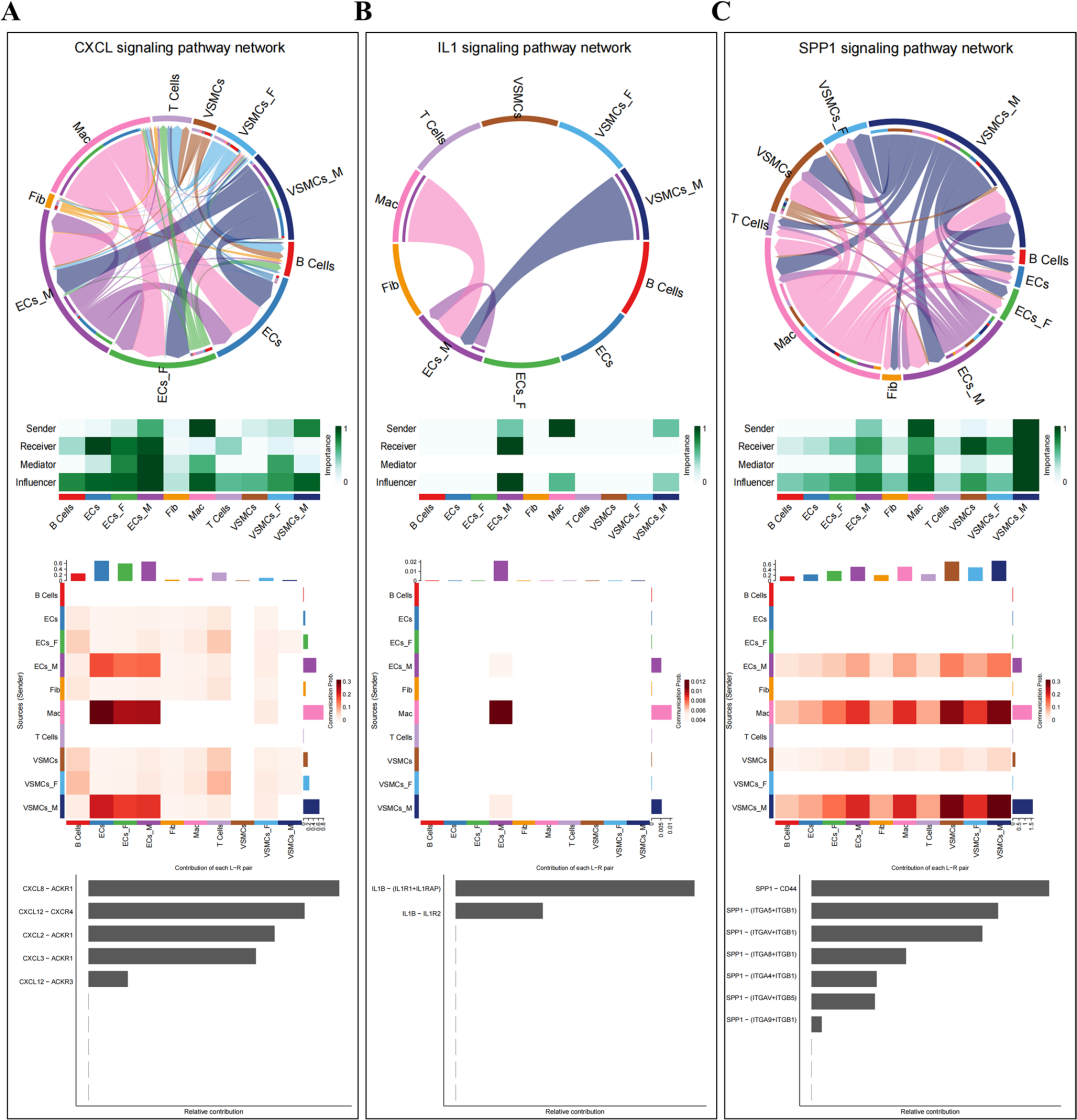
Major intercellular communication networks in human thoracic aortic dissection (TAD) tissue.** (A–C)** Cell–cell communication networks inferred from single-cell RNA sequencing data using CellChat analysis. Chord diagrams illustrate the interactions among major cell populations in the TAD microenvironment for three representative signaling pathways: CXCL **(A)**, IL1 **(B)**, and SPP1 **(C)**. Each segment represents a specific cell type, and connecting ribbons indicate ligand–receptor-mediated communication between sender and receiver cells. The width of each ribbon reflects the relative strength of the inferred signaling interaction. The heatmaps below the chord diagrams summarize the signaling roles of each cell population, including sender, receiver, mediator, and influencer, with color intensity representing the relative importance of each role within the pathway. The subsequent heatmaps show the communication probability between pairs of cell types, where rows represent source (sender) cells and columns represent target (receiver) cells. Color intensity indicates the strength of inferred signaling interactions. The bar plots at the bottom display the relative contributions of specific ligand–receptor pairs driving each signaling pathway. Abbreviations: ECs, endothelial cells; ECs_F, fibroblast-like endothelial cells; ECs_M, macrophage-like endothelial cells; Fib, fibroblasts; Mac, macrophages; VSMCs, vascular smooth muscle cells; VSMCs_F, fibroblast-like VSMCs; VSMCs_M, macrophage-like VSMCs

In this study, we provide the comprehensive characterization of phenotypic transdifferentiation in ECs and VSMCs in TAD, particularly the emergence of ECs_M and VSMCs_M. This phenotypic shift is accompanied by loss of structural identity in the intima and media and may represent a key pathological mechanism contributing to aortic wall tearing and dissection. Through cell–cell communication network analysis in TAD, we identified three major signaling axes by which macrophages regulate EC and VSMC behavior. CXCL signaling targeting ECs and ECs_M, involving: CXCL2/3/8–ACKR1, CXCL12–CXCR4, CXCL12–ACKR3 **(**Fig. 6A**)**. IL1B signaling targeting ECs_M, particularly: IL1B–(IL1R1 + IL1RAP)/IL1R2 **(**Fig. 6B**)**. SPP1 (osteopontin, OPN) signaling targeting ECs_M and VSMCs_M, including: SPP1–CD44, SPP1–(ITGA5 + ITGB1), SPP1–(ITGAV+ITGB1) **(**Fig. 6C**)**.

**Fig. 7:**
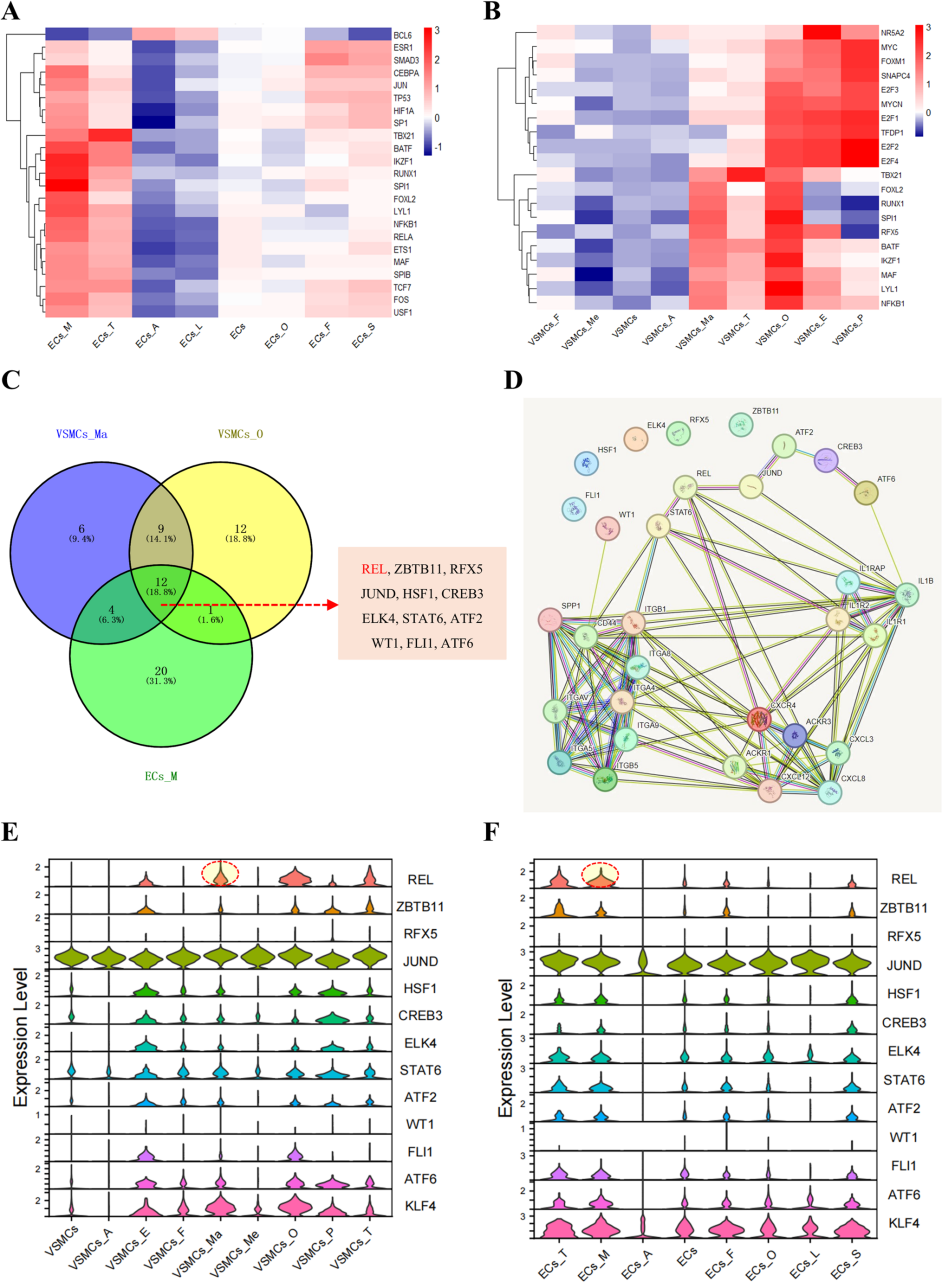
Regulatory network through which macrophages drive pro-inflammatory phenotypic changes in endothelial cells (ECs) and vascular smooth muscle cells (VSMCs) in thoracic aortic dissection (TAD).** (A**,** B)** Heatmaps showing predicted key transcription factors (TFs) regulating each EC **(A)** and VSMC **(B)** subpopulation identified from the single-cell RNA sequencing dataset of human TAD tissue. Rows represent transcription factors and columns represent cell subpopulations. Color intensity indicates relative gene expression levels (scaled expression). **(C)** Venn diagram showing the overlap of upregulated transcription factors among the macrophage-like VSMC subpopulation (VSMCs_Ma), osteogenic-like VSMCs (VSMCs_O), and macrophage-like endothelial cells (ECs_M). Shared TFs were selected as candidate regulators potentially involved in macrophage-driven inflammatory phenotypic transitions. **(D)** Protein–protein interaction (PPI) network illustrating interactions between the identified transcription factors and key signaling molecules involved in CXCL, interleukin-1 (IL-1), and secreted phosphoprotein 1 (SPP1, also known as osteopontin) signaling pathways. Nodes represent proteins and edges represent predicted or experimentally validated interactions. **(E**,** F)** Violin plots showing the expression levels of the candidate transcription factors across VSMC **(E)** and EC **(F)** subpopulations. The width of each violin represents the distribution of gene expression levels within the corresponding cell population

**Fig. 8:**
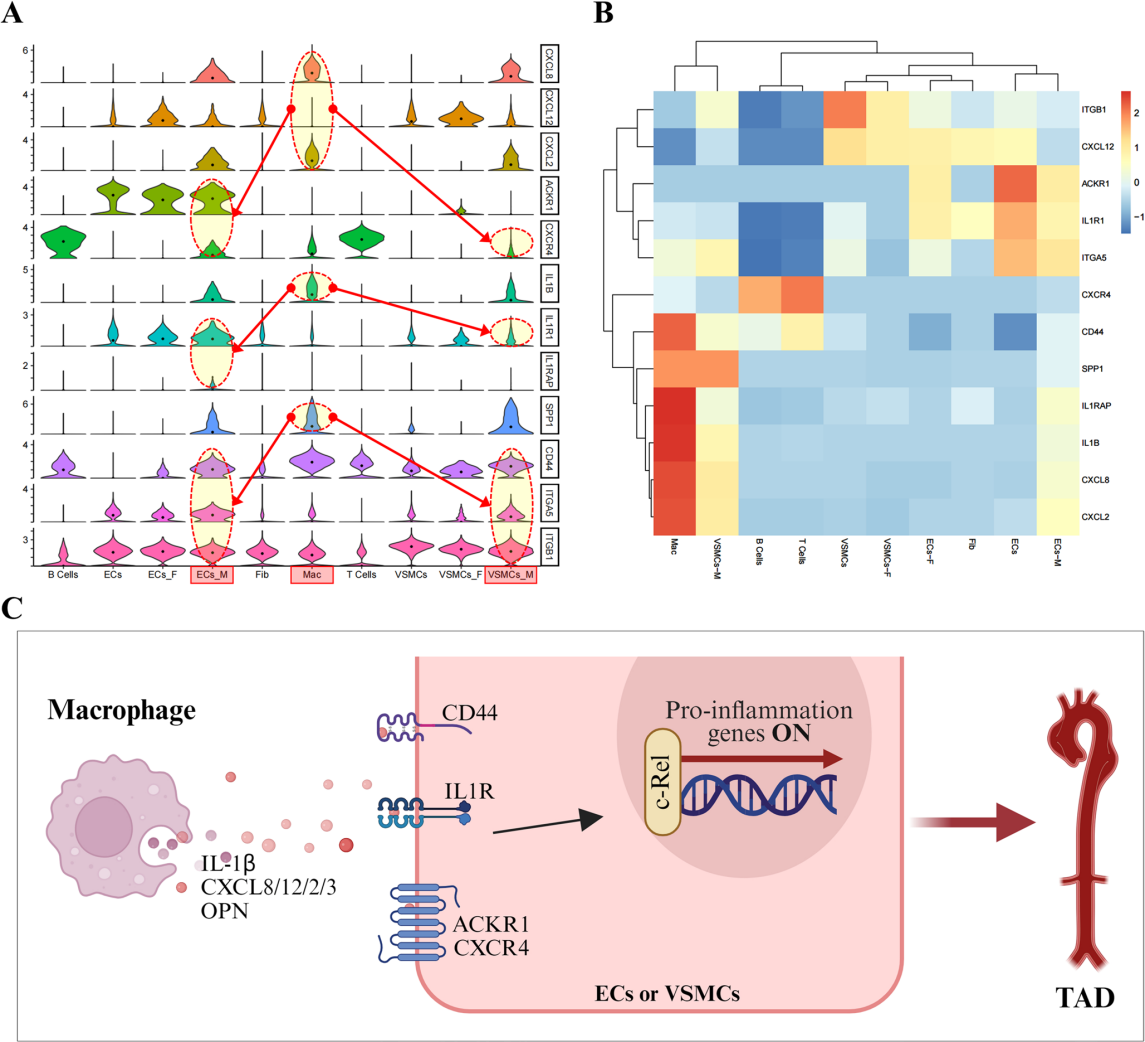
Macrophage-mediated inflammatory signaling in thoracic aortic dissection (TAD).** (A)** Violin plots showing the expression patterns of selected inflammatory signaling molecules across major vascular and immune cell populations, including B cells, endothelial cells (ECs), fibroblasts (Fib), macrophages (Mac), T cells, and vascular smooth muscle cells (VSMCs). Red dashed circles highlight cell populations with relatively high expression levels, suggesting potential cellular sources or targets of these inflammatory mediators. **(B)** Heatmap illustrating the relative expression levels of inflammatory signaling genes across cell subpopulations. Hierarchical clustering identifies distinct expression patterns among macrophages, ECs, and VSMC subsets. Genes associated with chemokine signaling, interleukin pathways, and adhesion molecules exhibit enriched expression in macrophages and specific vascular cell subsets. **(C)** Schematic diagram summarizing the proposed mechanism of macrophage-vascular cell communication in TAD. Activated macrophages release pro-inflammatory mediators such as IL-1β, CXCL8/12/2/3, and osteopontin (OPN, SPP1), which interact with receptors including CD44, IL1R, ACKR1, and CXCR4 on ECs or VSMCs. These signals activate downstream transcriptional regulators (e.g., c-Rel), leading to the upregulation of pro-inflammatory genes and contributing to the progression of thoracic aortic dissection

We further analyzed TFs associated with each EC and VSMC subpopulation **(**Fig. 7A, B**)**. Notably, VSMCs_Ma, VSMCs_O, and ECs_M shared 12 core TFs: REL, ZBTB11, RFX5, JUND, HSF1, CREB3, ELK4, STAT6, ATF2, WT1, FLI1, and ATF6 **(**Fig. 7C**)**. Because VSMCs_O express partial macrophage marker genes, they were included in the analysis. Subsequently, we performed protein–protein interaction (PPI) analysis integrating the core genes from the identified signaling pathways and the 12 shared TFs. REL (c-Rel), a member of the NF-κB family, emerged as a central hub at the intersection of all three macrophage-mediated signaling axes **(**Fig. 7D**)**. Expression patterns of all key genes were visualized **(**Fig. 7E, F**)**. Together, these findings delineate a unified mechanistic model: inflammatory macrophages release IL1B, CXCL2/3/8/12, and SPP1 to engage ECs and VSMCs **(**Fig. 8A, B**)**. This induces nuclear translocation of the transcription factor REL, which activates downstream pro-inflammatory gene expression programs, thereby driving phenotypic switching and promoting TAD progression **(**Fig. 8C**)**.

## Discussion

Using scRNA-seq data derived from human aortic tissues, we uncovered both shared and distinct features of vascular remodeling and cellular heterogeneity between AA and AD, providing several novel insights. First, we observed significant differences in cellular composition among AA and AD samples. In TAD, there was a notable increase in the proportion of intrinsic vascular wall cells (VSMCs and ECs) whereas their proportions were markedly reduced in both TAA and AAA. In AAA-rupture, only a small number of fibroblasts were present, with the remaining cells largely consisting of immune populations. Moreover, macrophages were significantly enriched in TAD and TAA, whereas T cells were predominantly increased in AAA, TAA, and AAA-rupture. Notably, B cells were highly upregulated in AAA, particularly in AAA-rupture. In murine AA models, we confirmed a progressive reduction of VSMCs during disease development, especially in the context of AAA rupture. Lineage-tracing experiments further demonstrated a gradual loss of VSMC markers, supporting the notion that VSMC phenotypic transdifferentiation represents an intermediate stage of disease progression, ultimately leading to cell death and aortic wall rupture. Next, we performed the subcluster annotation of ECs and VSMCs in TAD, and validated the presence of EC and VSMC subpopulations with macrophage-like phenotypes in a TAD mouse model. Finally, intercellular communication analysis revealed that macrophages induce nuclear translocation of REL through SPP1, IL1B, and CXCL signaling pathways, thereby promoting transcription of pro-inflammatory genes in ECs and VSMCs.

In recent years, VSMC phenotypic transdifferentiation and inflammation have emerged as major research foci in the pathogenesis of AAD. With the widespread application of single-cell sequencing and lineage-tracing technologies, phenotypic switching of VSMCs has been recognized as a hallmark of AAD [10, 30, 31, 36]. Under stress conditions, VSMCs lose their contractile phenotype and adopt alternative phenotypes, including fibroblast-like, myofibroblast-like, macrophage-like, osteogenic-like, and others [31, 37–39]. It is important to note that the direction of transdifferentiation may vary depending on the stage of disease progression, and different phenotypic shifts exert distinct effects on the vascular wall. Transition toward fibroblast-, myofibroblast-, or proliferative-like phenotypes may contribute to aortic remodeling and enhance structural support, but can also lead to ECM accumulation and vessel dilation [40]. In contrast, transdifferentiation into inflammatory or pro-death phenotypes reduces aortic compliance and impairs vascular function. In our scRNA-seq analysis of human AAA-rupture samples, intrinsic vascular wall cells were nearly absent, with most cells comprising immune populations. Consistently, our murine model of AAA rupture confirmed a marked depletion of VSMCs within the aortic wall. Furthermore, lineage-tracing studies in AAA mice revealed that VSMC phenotypic switching may represent a transitional state, culminating in the death of VSMCs across various phenotypes, ultimately contributing to structural failure and vessel rupture.

AA and AD share many pathogenic features and are frequently investigated collectively as a single entity (AAD) particularly in animal studies [41–44]. They exhibit common risk factors such as aging, hypertension, trauma, and atherosclerosis [8, 44], as well as similar injury mechanisms, including VSMC phenotypic switching, immune cell infiltration, and metabolic reprogramming [10, 19, 25, 45]. However, animal models often accelerate disease progression and fail to reflect the temporal dynamics, pathological phenotypes, and underlying mechanistic differences between AA and AD in human populations [13, 35, 46, 47]. AD typically has an insidious onset, with patients presenting acutely due to sudden tearing chest or back pain, by which time intimal and medial disruption of the aorta has already occurred [9, 48]. In contrast, AA is usually asymptomatic and often discovered incidentally during routine imaging examinations; acute pain and hemodynamic collapse are indicative of rupture or impending rupture [49, 50]. AD resembles a seemingly tranquil snow-covered mountain that suddenly collapses in an avalanche-like event with extremely high mortality [14], whereas AA follows a “dripping water wears away the stone” pattern of slow evolution, in which the imbalance between proliferative repair and decreased aortic compliance eventually leads to rupture through cumulative damage. In our study, we observed an increased proportion of intrinsic vascular cells (ECs and VSMCs) in TAD, whereas their proportions were markedly reduced in AA, especially in AAA-rupture. Moreover, in a TAD mouse model, we identified EC and VSMC subpopulations undergoing transdifferentiation into macrophage-like phenotypes. These ECs_M and VSMCs_M subsets exhibit macrophage-like migratory behavior, which not only disrupts the endothelial barrier but also compromises the structural integrity and tight connectivity between the intima and adventitia. This mechanical discontinuity facilitates the penetration of pulsatile blood flow, ultimately resulting in aortic tearing.

In the study of AD, the role of ECs is increasingly recognized as essential. In our analysis, the proportion of ECs was reduced in TAA and AAA, suggesting that EC loss occurs during disease progression and that ECs may play a more prominent role in the early stages of disease. In contrast, ECs were enriched in TAD, where multiple distinct EC subpopulations could be robustly identified through clustering analysis. A previous study demonstrated that, at early time points (days 5 and 10) in a murine model of AAD, tight junction function in ECs was disrupted. This compromise of barrier integrity promoted immune cell infiltration and blood flow penetration and represented an early event preceding AAD formation [51]. Luo et al. revealed that ECs contribute to AAD progression by forming a HDAC1-ZEB2-NuRD complex, which regulates protein S-sulfhydration [43]. Wang et al. identified a subpopulation of ECs with high expression of ACKR1 that mediates macrophage migration and pro-inflammatory polarization via the ACKR1/NF-κB/SPP1 signaling axis, playing a critical role in TAD progression [26]. Furthermore, in FBN1-deficient mice, interactions between ECs and macrophages/monocytes were shown to facilitate the onset of early-stage AD [27]. Collectively, these findings highlight the emerging importance of ECs in the pathogenesis of AAD, particularly during the early stages of disease development.

In this study, we found that macrophages activate the NF-κB signaling pathway through SPP1, IL1B, and CXCL signaling, thereby promoting the transdifferentiation of ECs and VSMCs into macrophage-like phenotypes. Roy-Chowdhury et al. demonstrated in atherosclerotic disease that CD16⁺ monocytes enhance phosphorylation of STAT1 and NF-κB p65 in ECs, leading to upregulation of CX3CL1 and IL-1β, along with increased expression of various CCL and CXCL chemokines and adhesion molecules such as ICAM1 and VCAM1, which facilitate leukocyte patrolling and adhesion [52]. IL-1β, a key pro-inflammatory cytokine in the IL1B signaling pathway, plays a crucial role in inducing phenotypic changes in ECs, fibroblasts, and monocytes [53–55]. Additionally, SPP1⁺ macrophages may exert dual roles in aortic disease by contributing to both inflammation and fibrosis [56–58].

This study has several limitations. First, the phenotypic transitions of ECs and VSMCs were inferred primarily from single-cell transcriptomic analyses and were not validated using lineage-tracing mouse models, mainly due to the limited availability of genetically engineered mice in our current experimental resources. Therefore, the cellular plasticity and transdifferentiation trajectories proposed in this study should be interpreted with caution. Second, although our analyses identified key transcriptional regulators and signaling pathways potentially involved in these phenotypic transitions, the precise molecular mechanisms driving EC and VSMC transdifferentiation remain to be fully elucidated. Future studies incorporating in vitro functional experiments and genetically engineered mouse models will be necessary to validate these cellular transitions and to further dissect the underlying molecular mechanisms.

## Conclusions

In summary, our single-cell transcriptomic analysis of human aortic tissues revealed both shared and disease-specific patterns of cellular remodeling and heterogeneity in AA and AD. We highlighted the dynamic evolution of immune and structural cell populations across distinct types of aortic disease, particularly noting that rupture-AAA is characterized by a predominance of immune cells and a marked loss of VSMCs. Lineage-tracing and murine model validation further confirmed that phenotypic switching and the eventual depletion of resident vascular cells are key events in disease progression. Importantly, in AD, macrophage-mediated signaling emerged as a central driver of pro-inflammatory reprogramming in ECs and VSMCs. These findings provide novel insights into the mechanisms underlying aortic wall instability and offer promising directions for the identification of potential therapeutic targets.

## Supplementary Information

Below is the link to the electronic supplementary material.

Supplementary Material 1 (XLSX 201 KB)

Supplementary Material 2 (XLSX 50.8 KB)

Supplementary Material 3 (XLSX 128 KB)

Supplementary Material 4 (PDF 29.5 MB)

## Data Availability

The datasets analysed during the current study are available in the public Gene Expression Omnibus database, https://www.ncbi.nlm.nih.gov/geo. The data used during the current study are available from the corresponding author on reasonable request.

## Abbreviations

AA: Aortic aneurysm
AD: Aortic dissection
scRNA-seq: Single-cell RNA sequencing
TAD: Thoracic aortic dissection
TAA: Thoracic aortic aneurysm
AAA: Abdominal aortic aneurysm
VSMCs: Vascular smooth muscle cells
ECs: Endothelial cells
ECM: Extracellular matrix
AAD: Aortic aneurysmal disease
NETs: Neutrophil extracellular traps
GEO: Gene expression omnibus
HVGs: Highly variable genes
PCA: Principal component analysis
SNN: Shared nearest neighbor
UMAP: Uniform manifold approximation and projection
DCs: Dendritic cells
MCs: Mast cells
NK cells: Natural killer cells
KEGG: Kyoto Encyclopedia of Genes and Genomes
GO: Gene Ontology
BP: Biological processes
CC: Cellular components
MF: Molecular functions
TF: Transcription factor
BAPN: β-aminopropionitrile
PPE: Porcine pancreatic elastase
HE: Hematoxylin and eosin
EVG: Elastic Van Gieson
α-SMA: α-smooth muscle actin
IF: Immunofluorescence
