# Supplementary material for: Erosion or Explosion: Integrated Single-Cell Transcriptomics Reveals Cellular Heterogeneity in Aortic Aneurysm and Dissection
Source: Inflammation. 2026 May 12;49(1):159. doi: 10.1007/s10753-026-02523-5 (PMC13337744; doi:10.1007/s10753-026-02523-5)
Supplement: Supplementary file 4 — Supplementary Material 4 (PDF 29.5 MB) [file 10753_2026_2523_MOESM4_ESM.pdf]

Supplementary Figures

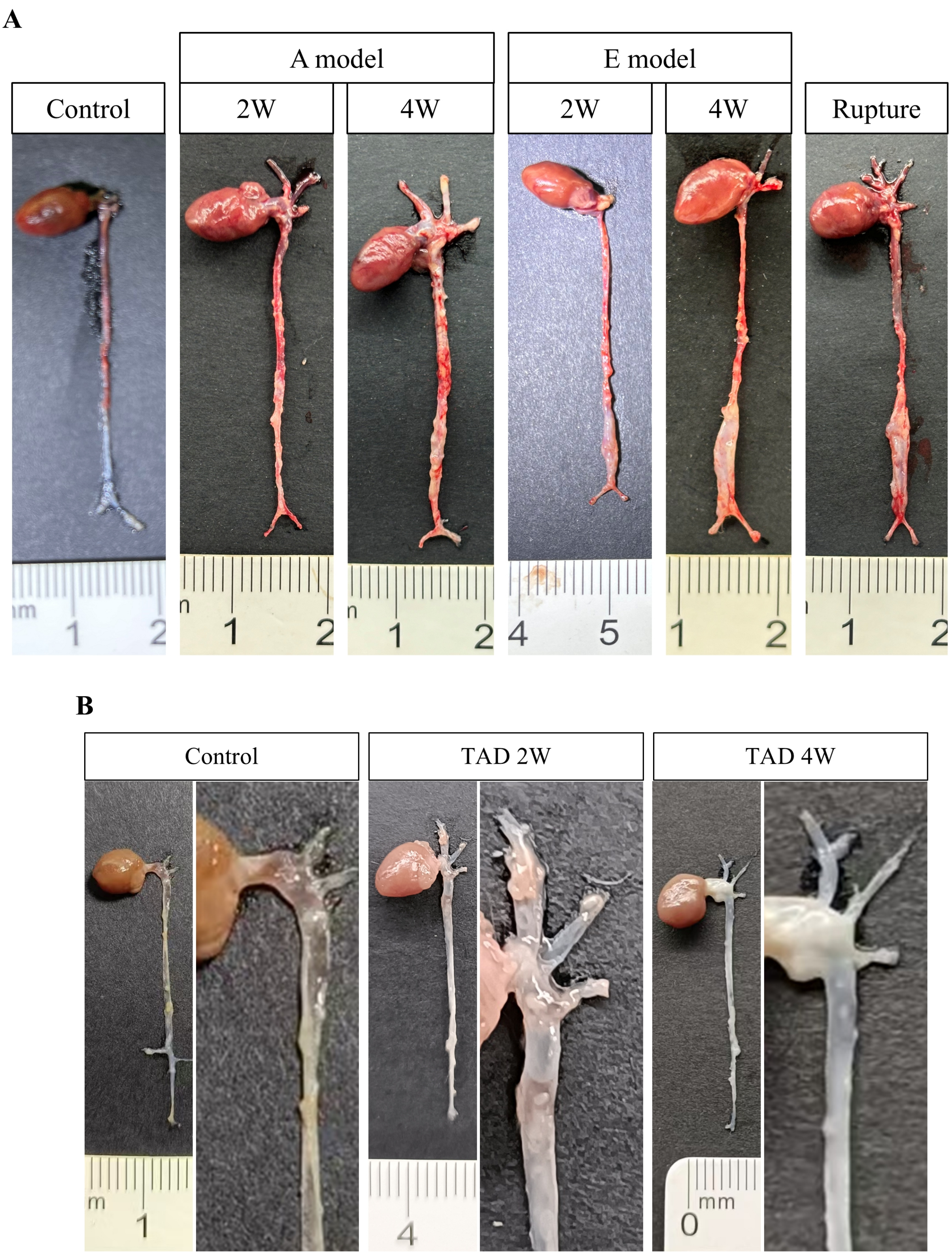

Supplementary Fig. 1. Gross morphology of mouse aortas in experimental aortic aneurysm and thoracic aortic dissection models. (A) Representative gross images of aortas from mouse abdominal aortic aneurysm (AA) models. A model indicates the angiotensin II (AngII)

infusion–induced aneurysm model, while E model indicates the elastase perfusion–induced aneurysm model. Aortas were harvested at 2 and 4 weeks after induction and compared with control aortas. A representative ruptured aneurysm is also shown. **(B)** Representative gross images of aortas from the mouse thoracic aortic dissection (TAD) model at 2 and 4 weeks after induction, compared with control aortas. Rulers indicate millimeter scale.

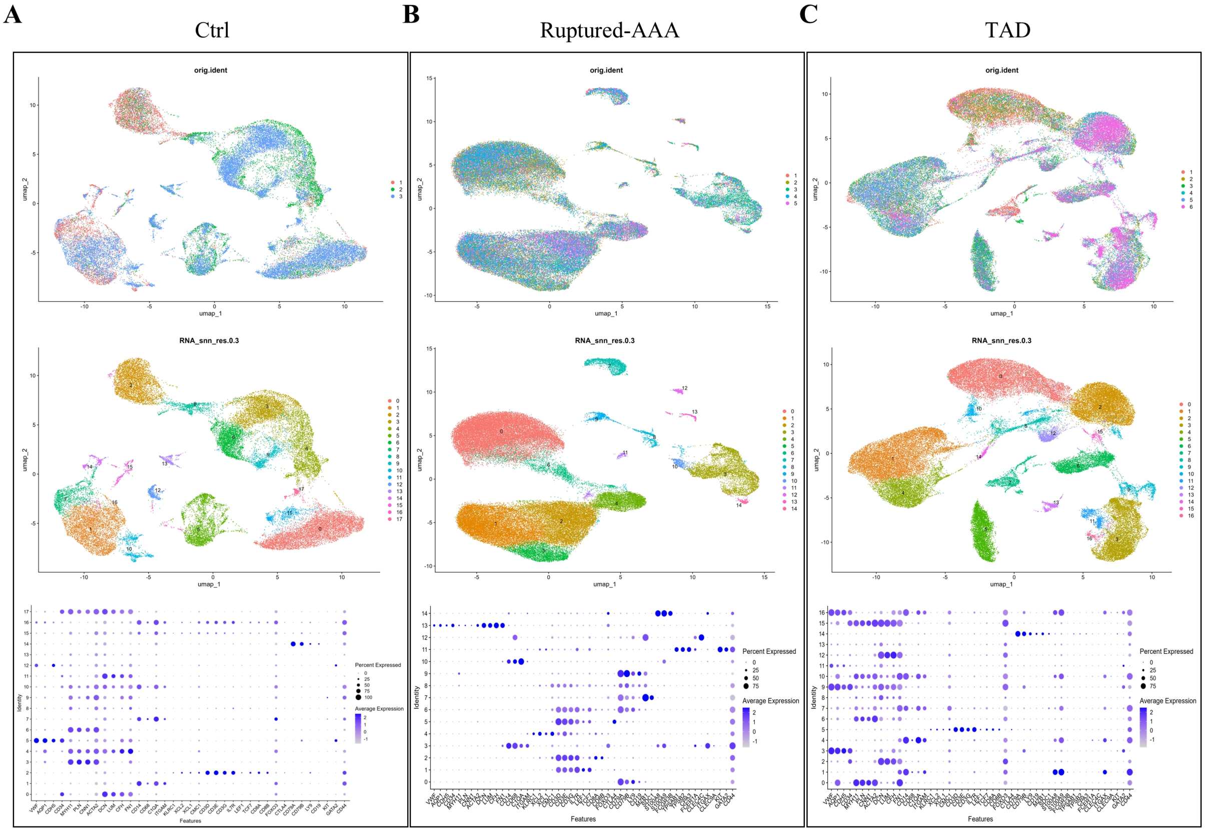

Supplementary Fig. 2. Single-cell clustering and marker gene annotation of human aortic tissues. (A) UMAP visualization of single-cell transcriptomic profiles from normal control aortic tissue (Ctrl). The upper panel shows cell clustering, and the middle panel shows annotated cell populations based on canonical marker genes. The lower panel displays a dot plot of representative marker genes used for cell-type identification. **(B)** UMAP visualization of single-cell transcriptomic profiles from ruptured abdominal aortic aneurysm (AAA) tissue. The upper panel shows unsupervised clustering, and the middle panel shows annotated cell populations. The lower panel presents marker gene expression across identified cell clusters. **(C)** UMAP visualization of single-cell transcriptomic profiles from thoracic aortic dissection (TAD) tissue. The upper panel shows unsupervised clustering, the middle panel shows cell-type annotation based on marker genes, and the lower panel presents the dot plot of representative marker genes across clusters. Dot size represents the percentage of cells expressing the gene, and color intensity indicates the average expression level.

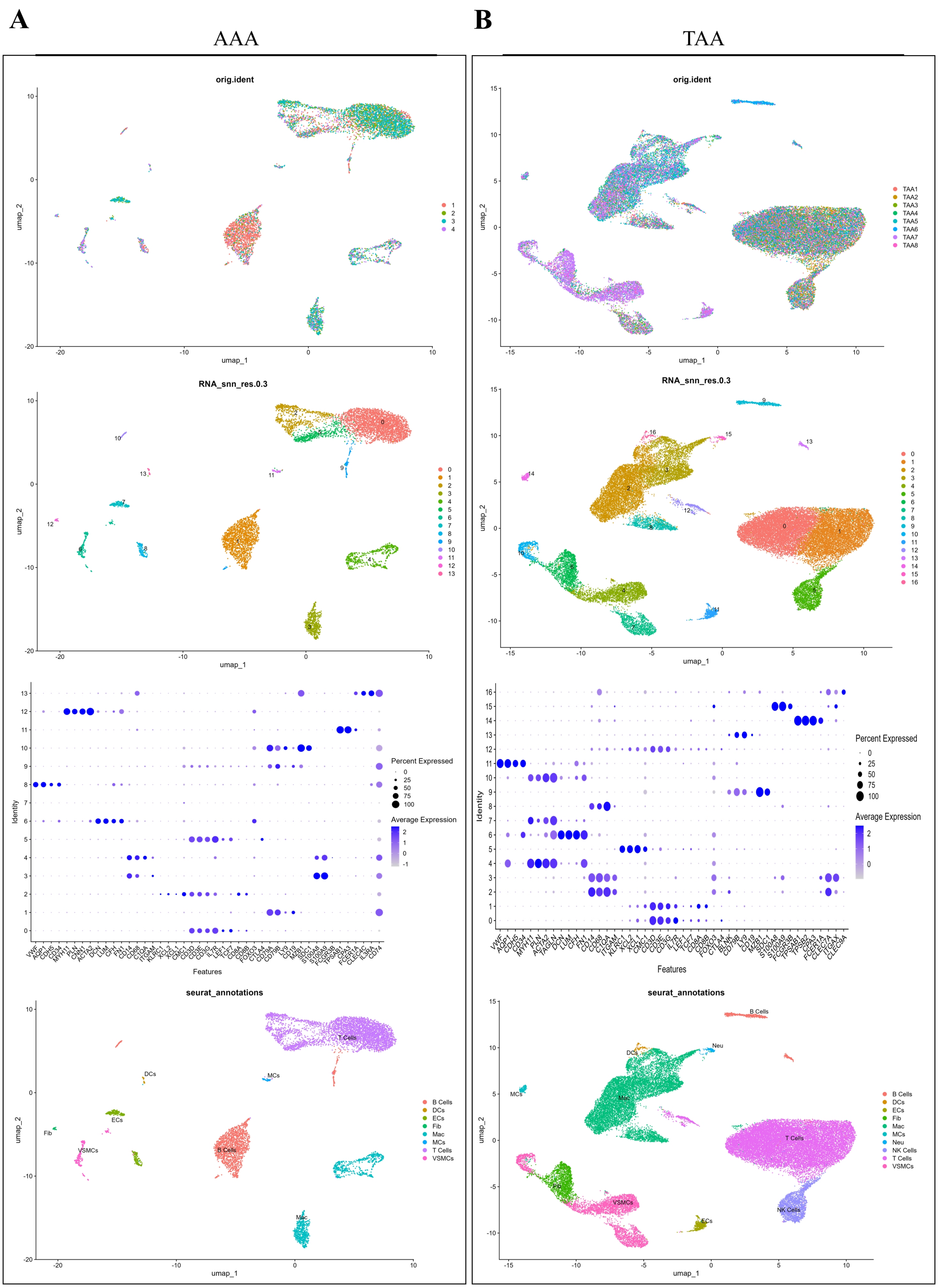

Supplementary Fig. 3. Single-cell clustering and marker gene annotation of human abdominal aortic aneurysm (AAA) and thoracic aortic aneurysm (TAA) tissues. (A) UMAP visualization of single-cell transcriptomic profiles from AAA tissue. The top panel shows the distribution of cells by sample identity (orig.ident). The second panel shows unsupervised clustering results (resolution

= 0.3). The third panel presents a dot plot of representative marker genes used for cell-type identification. The bottom panel shows the annotated cell populations based on canonical marker genes. **(B)** UMAP visualization of single-cell transcriptomic profiles from TAA tissue. The top panel shows cells colored by sample identity. The second panel displays unsupervised clustering results (resolution = 0.3). The third panel shows marker gene expression across clusters using a dot plot. The bottom panel presents the final cell-type annotation. Dot size represents the percentage of cells expressing each gene, and color intensity indicates the average expression level.

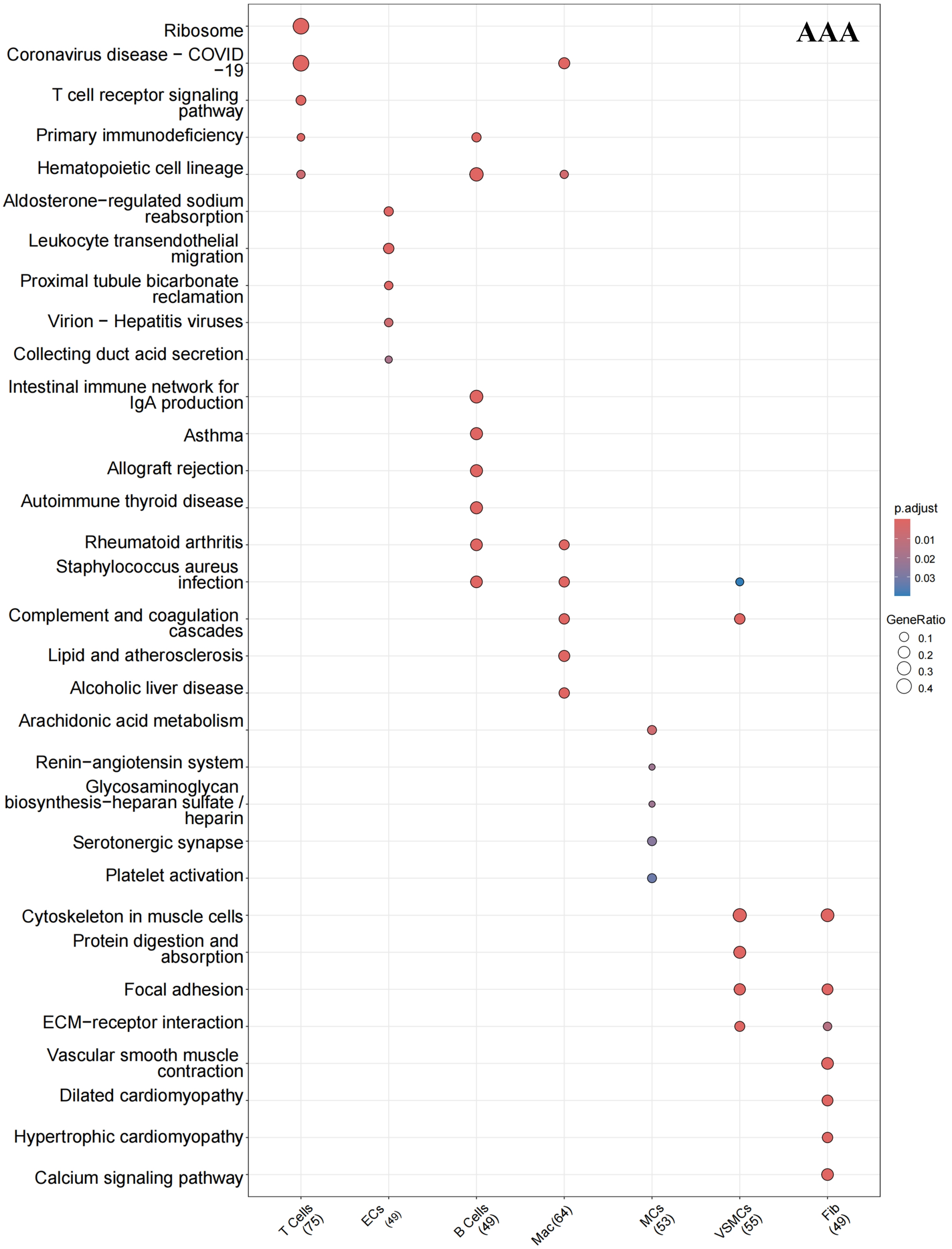

Supplementary Fig. 4. KEGG pathway enrichment analysis across major cell populations in abdominal aortic aneurysm (AAA). KEGG pathway enrichment analysis of differentially expressed genes in major cell populations identified in AAA tissue, including T cells, endothelial cells (ECs), B cells, macrophages (Mac), mast cells (MCs), vascular smooth muscle cells (VSMCs), and fibroblasts (Fib). Each dot represents a significantly enriched pathway. Dot size indicates the

gene ratio (GeneRatio), and color represents the adjusted p value (p.adjust).

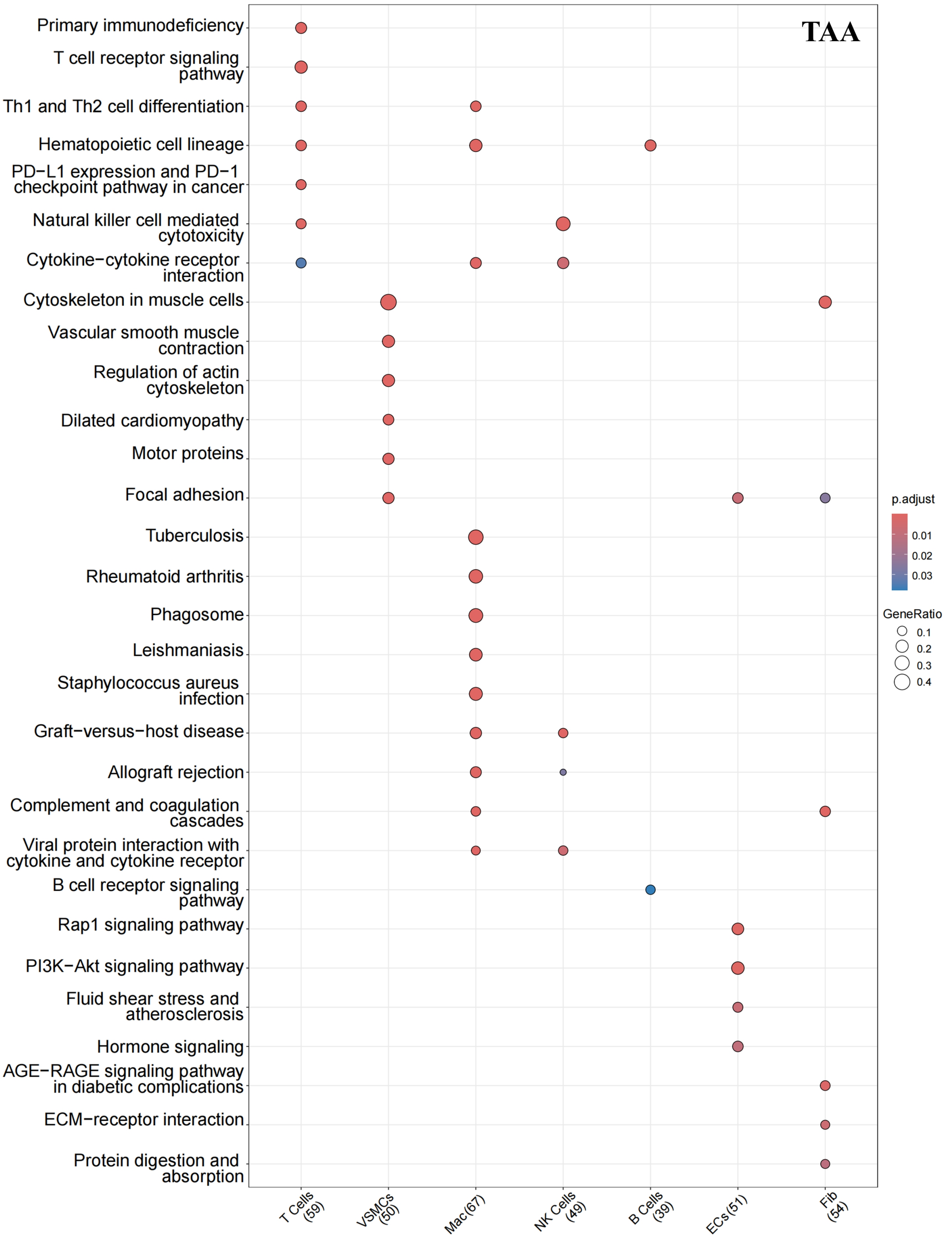

Supplementary Fig. 5. KEGG pathway enrichment analysis across major cell populations in thoracic aortic aneurysm (TAA). KEGG pathway enrichment analysis of differentially expressed genes in major cell populations identified in TAA tissue, including T cells, vascular smooth muscle cells (VSMCs), macrophages (Mac), natural killer (NK) cells, B cells, endothelial cells (ECs), and fibroblasts (Fib). Each dot represents a significantly enriched pathway. Dot size indicates the gene

ratio (GeneRatio), and color represents the adjusted p value (p.adjust).

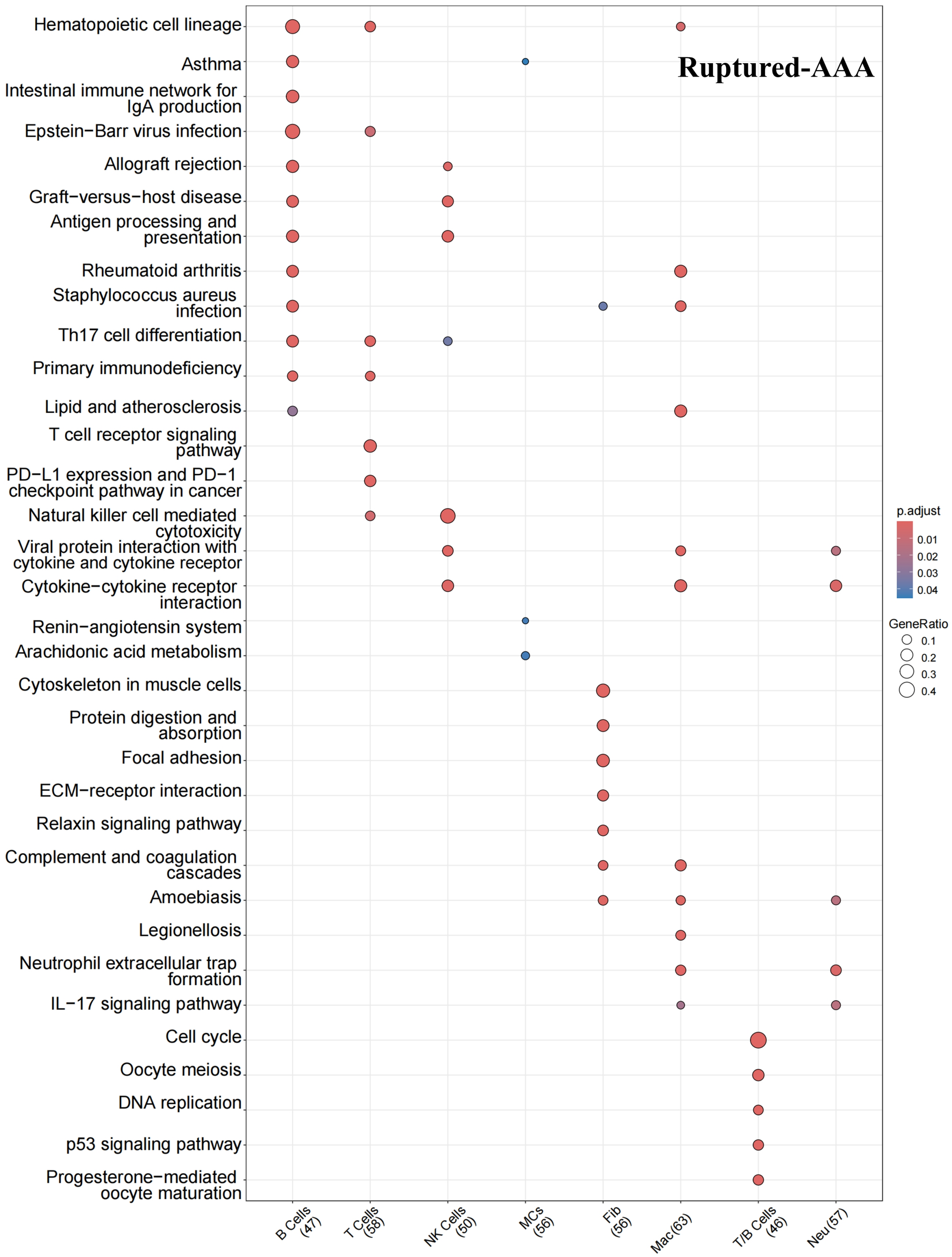

Supplementary Fig. 6. KEGG pathway enrichment analysis across major cell populations in ruptured abdominal aortic aneurysm (ruptured-AAA). KEGG pathway enrichment analysis of differentially expressed genes in major cell populations identified in ruptured-AAA tissue, including B cells, T cells, natural killer (NK) cells, mast cells (MCs), fibroblasts (Fib), macrophages (Mac), T/B cells, and neutrophils (Neu). Each dot represents a significantly enriched pathway. Dot size

indicates the gene ratio (GeneRatio), and color represents the adjusted p value (p.adjust).

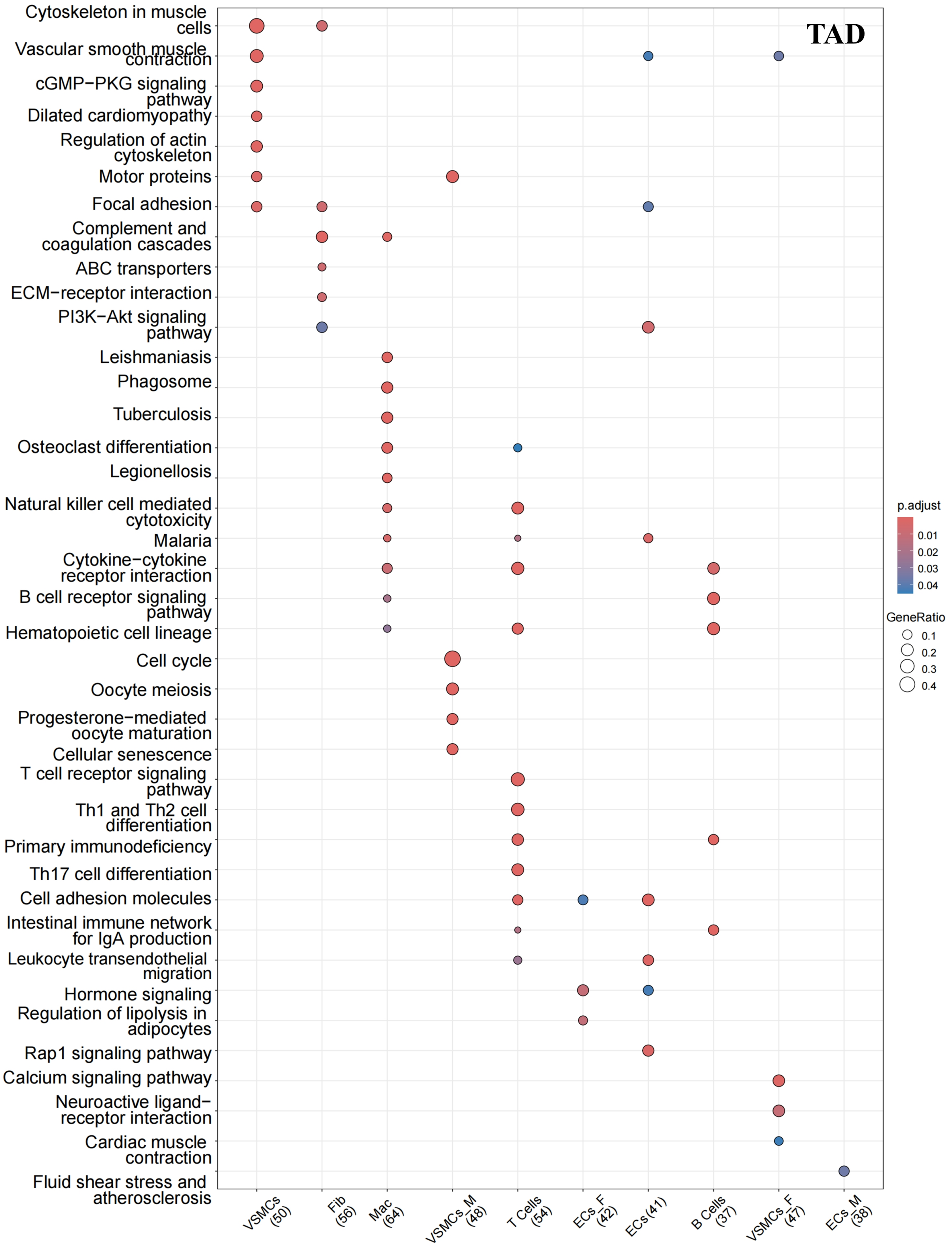

Supplementary Fig. 7. KEGG pathway enrichment analysis across major cell populations in thoracic aortic dissection (TAD). KEGG pathway enrichment analysis of differentially expressed genes in major cell populations identified in TAD tissue, including vascular smooth muscle cells (VSMCs), fibroblasts (Fib), macrophages (Mac), VSMC-M–like cells, T cells, endothelial cells (ECs), B cells, and neutrophils (Neu). Each dot represents a significantly enriched pathway. Dot

size indicates the gene ratio (GeneRatio), and color represents the adjusted p value (p.adjust).

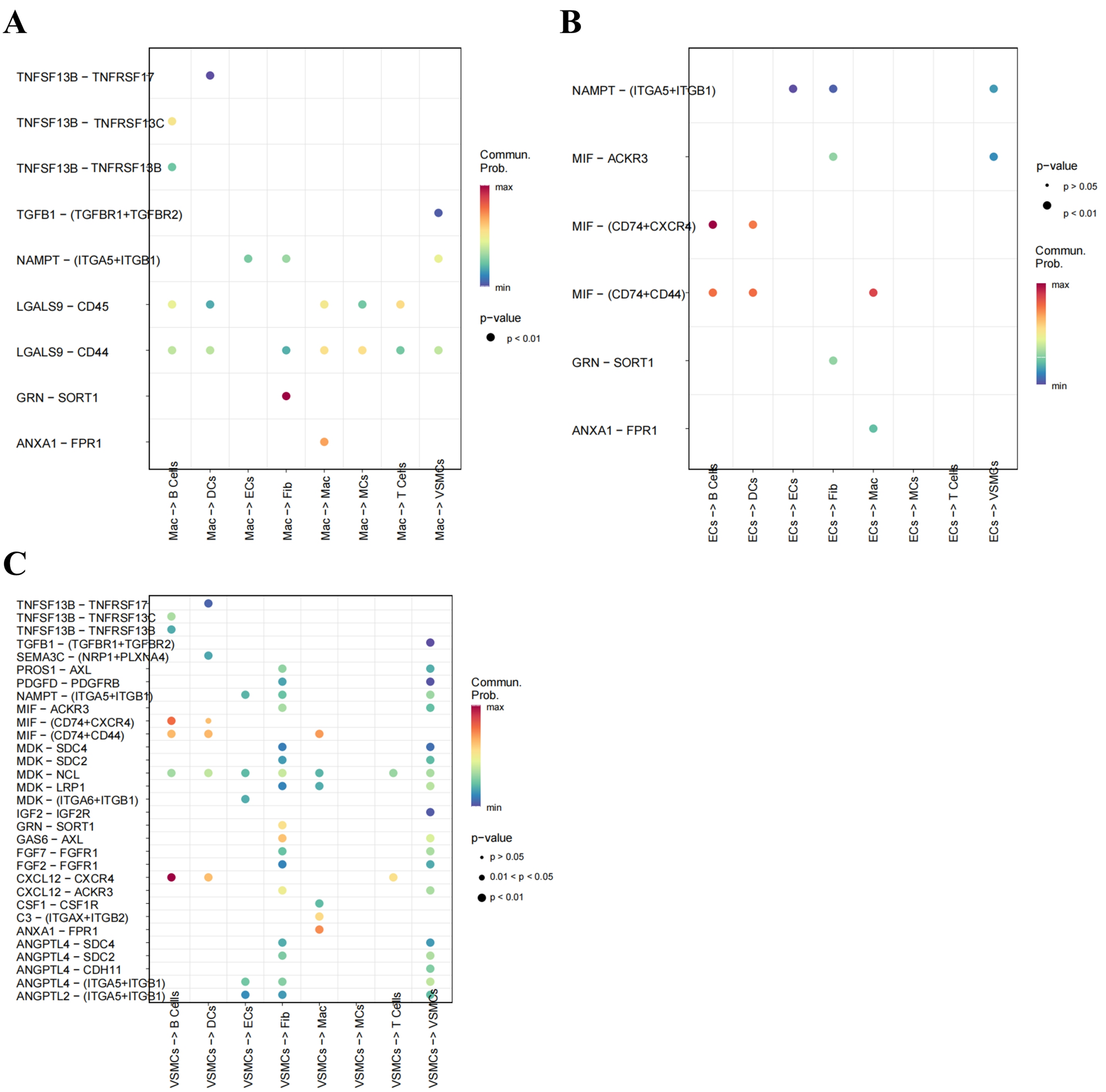

Supplementary Fig. 8. Intercellular communication analysis among major cell populations in human abdominal aortic aneurysm (AAA) tissue. (A) Predicted ligand–receptor interactions mediated by macrophages (Mac) with other cell populations in AAA tissue. **(B)** Predicted ligand–receptor interactions mediated by endothelial cells (ECs) with other cell populations. **(C)** Predicted ligand–receptor interactions mediated by vascular smooth muscle cells (VSMCs) with other cell populations. Each dot represents a ligand–receptor pair involved in intercellular communication between the indicated sender and receiver cell populations. Dot color indicates the communication probability, and dot size reflects the statistical significance of the interaction.

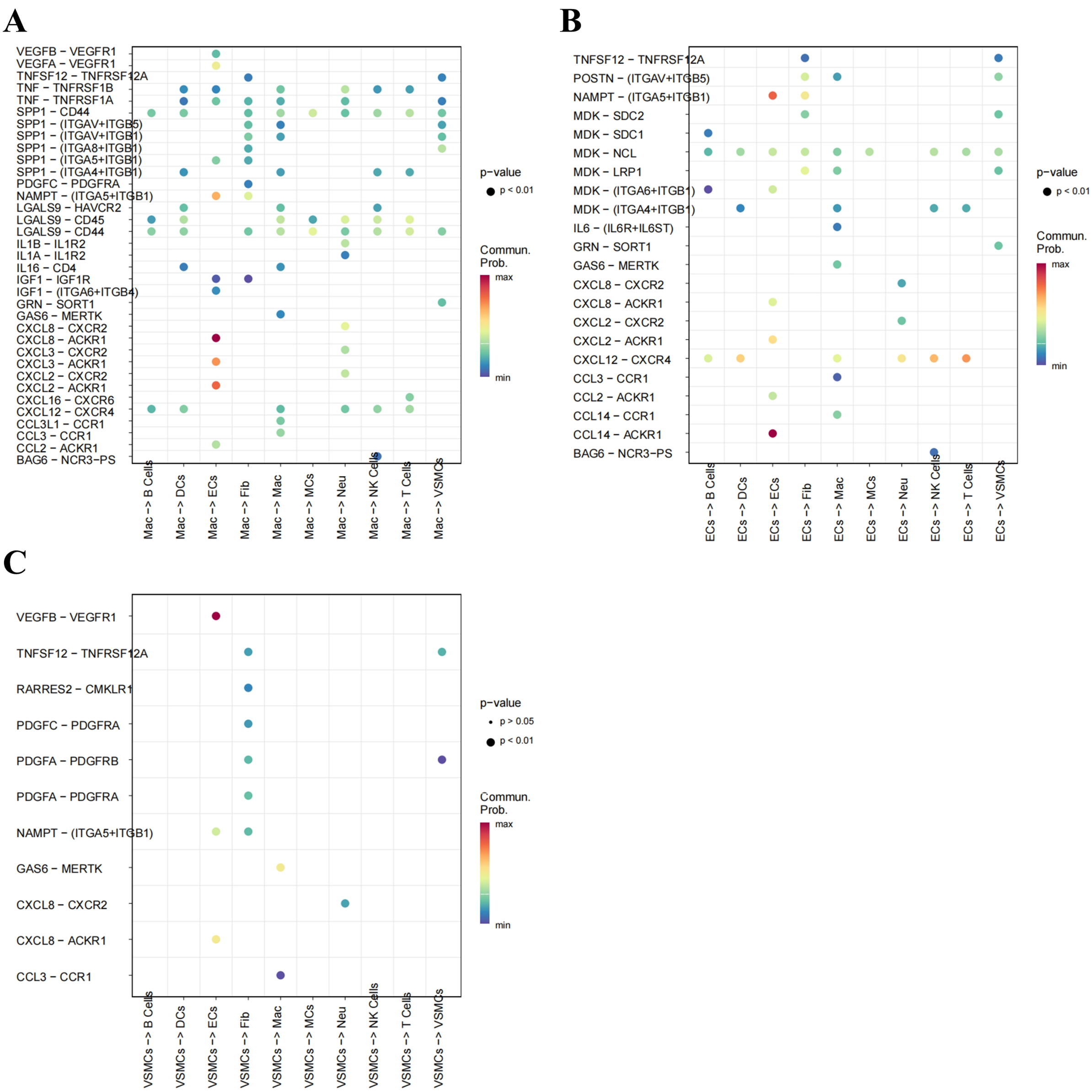

Supplementary Fig. 9. Intercellular communication networks among major cell populations in human thoracic aortic aneurysm (TAA) tissue. (A) Predicted ligand–receptor interactions mediated by macrophages (Mac) with other cell populations in TAA tissue. **(B)** Predicted ligand–receptor interactions mediated by endothelial cells (ECs) with other cell populations. **(C)** Predicted ligand–receptor interactions mediated by vascular smooth muscle cells (VSMCs) with other cell populations. Ligand–receptor interactions were inferred using the CellChat algorithm. Each dot represents a ligand–receptor pair involved in intercellular communication between the indicated sender and receiver cell populations. Dot color indicates the communication probability, and dot size represents the statistical significance of the interaction.

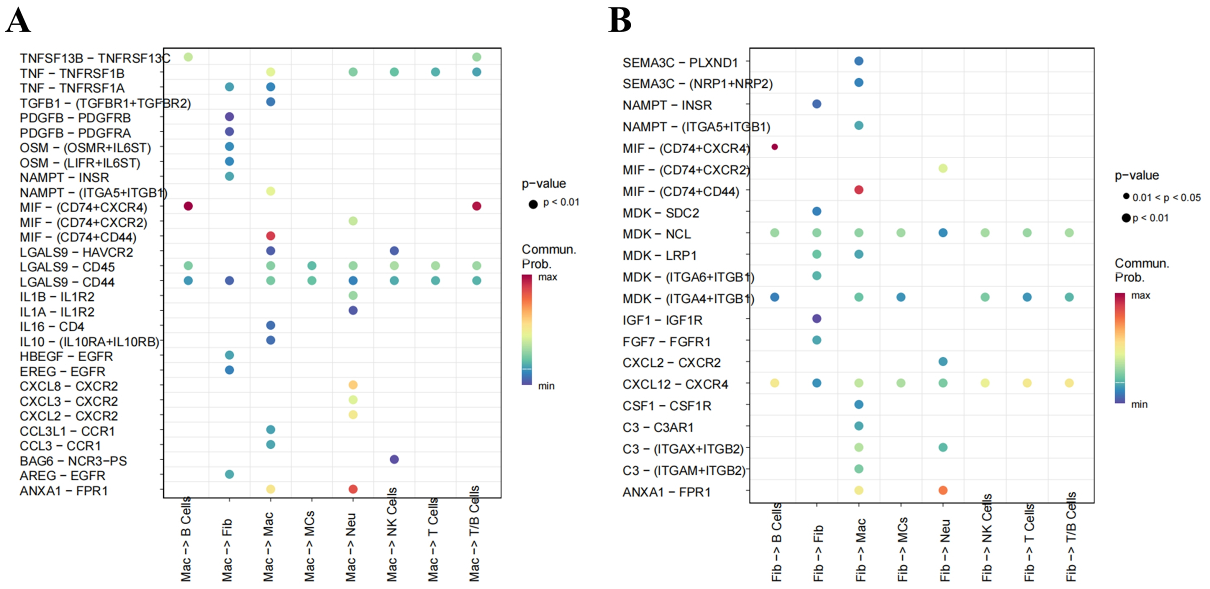

Supplementary Fig. 10. Intercellular communication networks among major cell populations in human ruptured abdominal aortic aneurysm (AAA) tissue. (A) Predicted ligand–receptor interactions mediated by macrophages (Mac) with other cell populations in ruptured AAA tissue. **(B)** Predicted ligand–receptor interactions mediated by fibroblasts (Fib) with other cell populations. Ligand–receptor interactions were inferred using the CellChat algorithm. Each dot represents a ligand–receptor pair involved in intercellular communication between the indicated sender and receiver cell populations. Dot color indicates the communication probability, and dot size represents the statistical significance of the interaction.

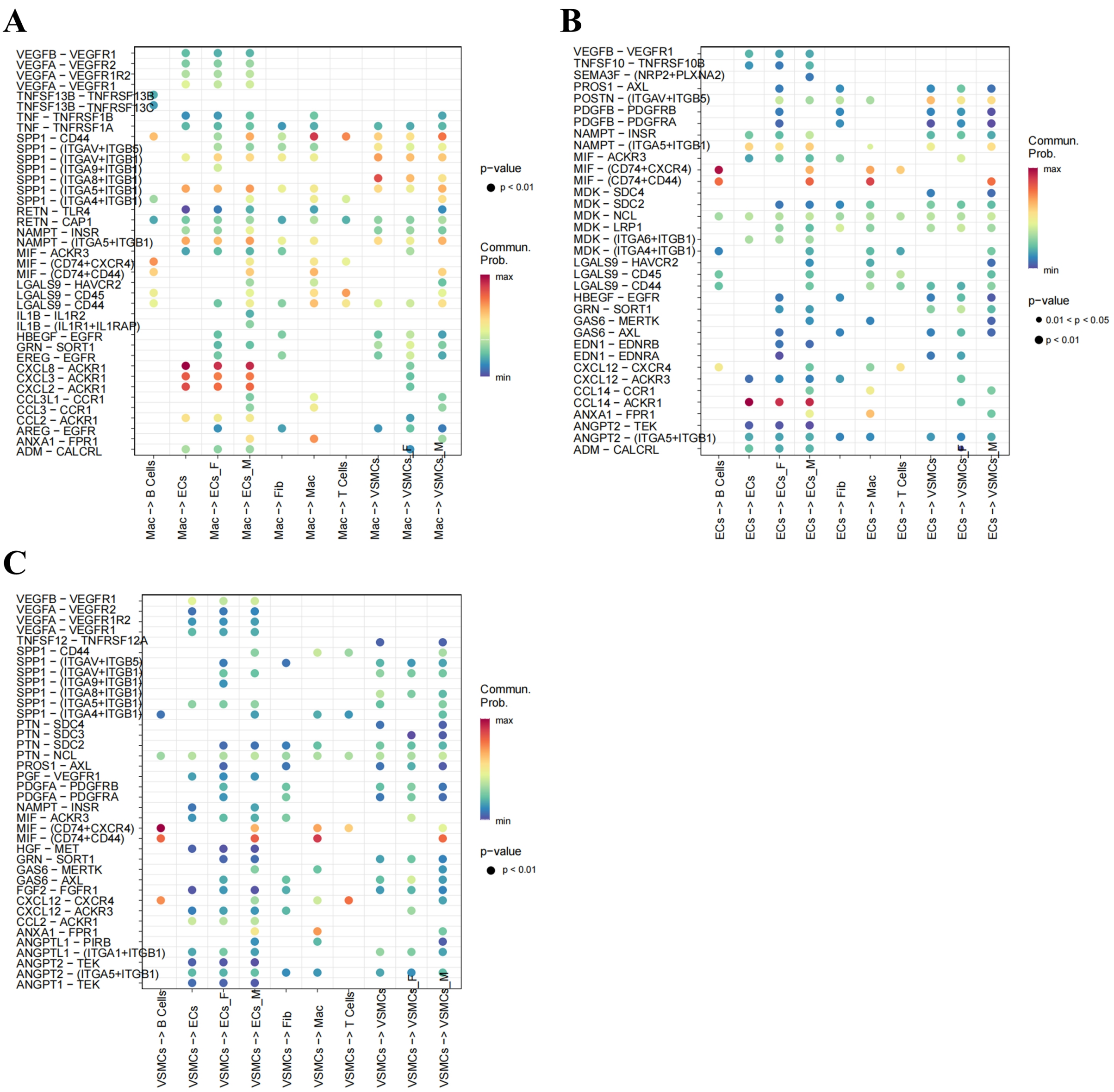

Supplementary Fig. 11. Intercellular communication networks among major cell populations in human thoracic aortic dissection (TAD) tissue. (A) Predicted ligand–receptor interactions mediated by macrophages (Mac) with other cell populations in TAD tissue. **(B)** Predicted ligand–receptor interactions mediated by endothelial cells (ECs) with other cell populations. **(C)** Predicted ligand–receptor interactions mediated by vascular smooth muscle cells (VSMCs) with other cell populations. Ligand–receptor interactions were inferred using the CellChat algorithm. Each dot represents a ligand–receptor pair involved in intercellular communication between the indicated sender and receiver cell populations. Dot color indicates the communication probability, and dot size represents the statistical significance of the interaction.

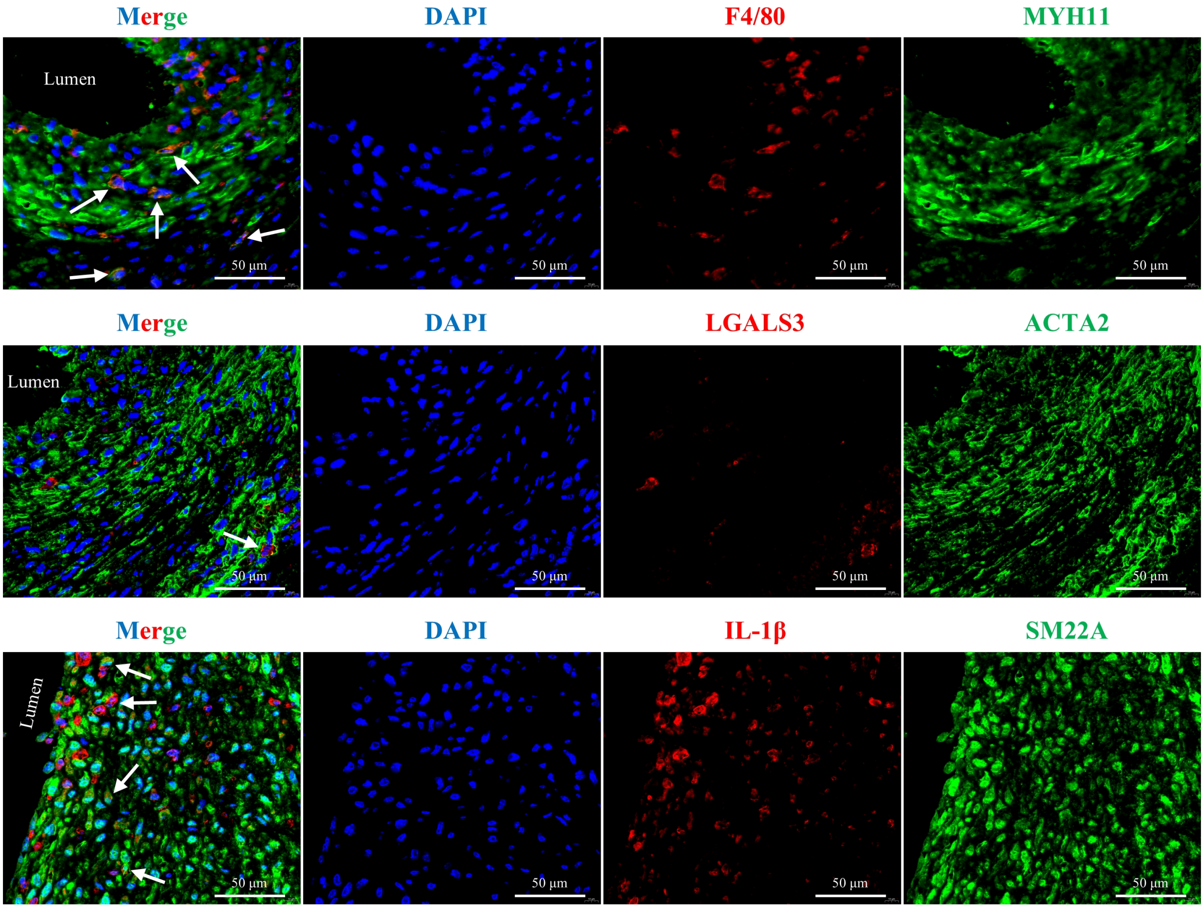

Supplementary Fig. 12. Immunofluorescence staining of macrophage and vascular smooth muscle cell markers in aortic tissues from the mouse abdominal aortic aneurysm (AAA) model. Representative immunofluorescence images of aortic sections from AAA mice showing the expression of macrophage and vascular smooth muscle cell markers. The upper panel shows F4/80 (red) and MYH11 (green) staining. The middle panel shows LGALS3 (red) and ACTA2 (green) staining. The lower panel shows IL-1β (red) and SM22A (green) staining. Nuclei were counterstained with DAPI (blue). Arrows indicate cells co-expressing macrophage markers and smooth muscle cell markers in the aortic wall. Scale bars, 50 μm.

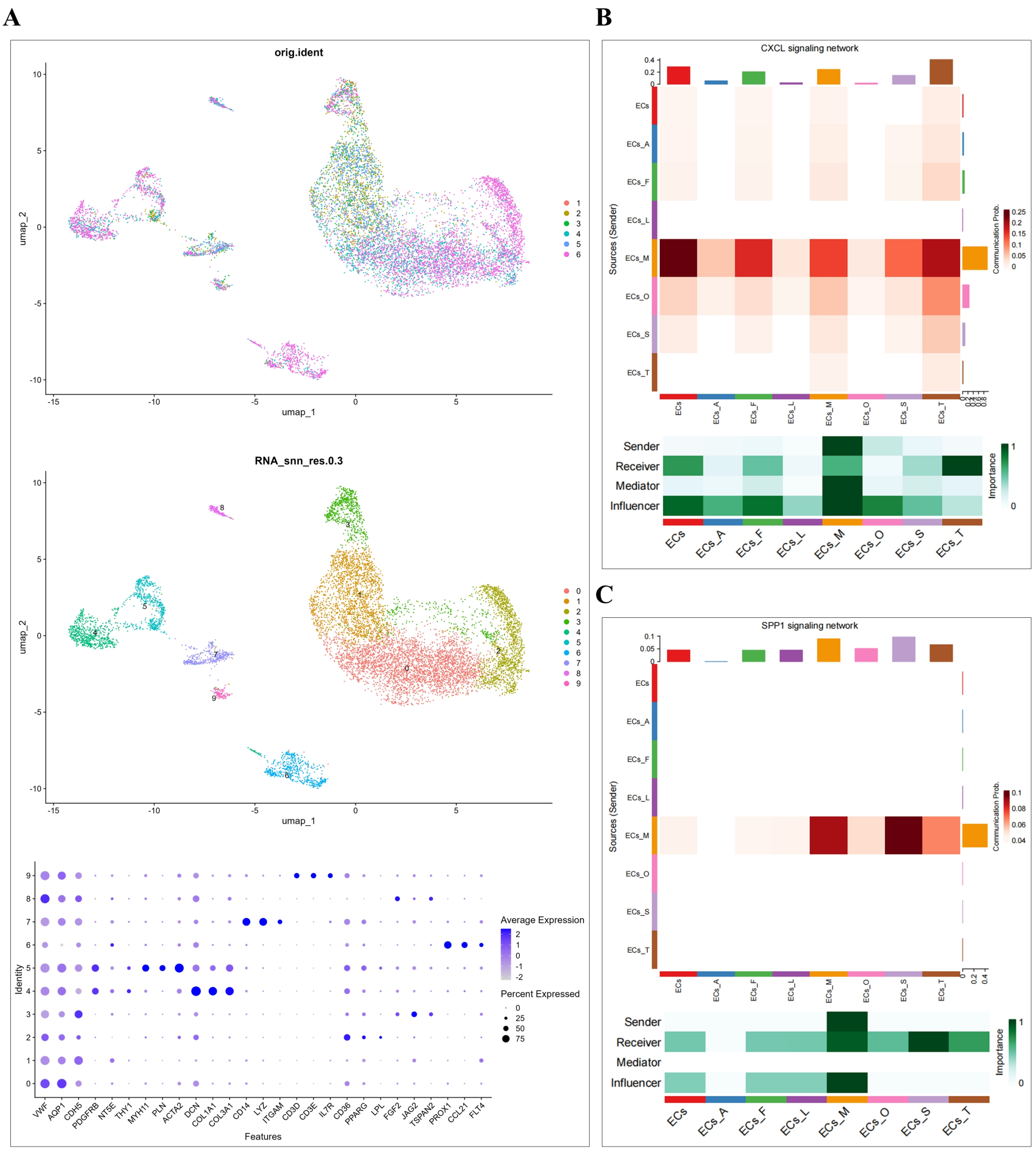

Supplementary Fig. 13. Endothelial cell (EC) subpopulation clustering and intercellular communication analysis in human thoracic aortic dissection (TAD) tissue. (A) UMAP visualization of endothelial cell (EC) subpopulations identified in TAD tissue. The upper panel shows cells colored by sample identity (orig.ident), and the middle panel shows unsupervised clustering results (resolution = 0.3). The lower panel presents a dot plot of representative marker genes used for EC subpopulation annotation. **(B)** Predicted CXCL signaling network among EC subpopulations inferred using the CellChat algorithm. Heatmaps indicate the communication probability between sender and receiver EC subpopulations, and the relative signaling roles of each EC subpopulation are summarized below. **(C)** Predicted SPP1 signaling network among EC

subpopulations inferred by CellChat. Heatmaps show the communication strength between EC subpopulations, and the signaling roles of each EC subtype are summarized below.

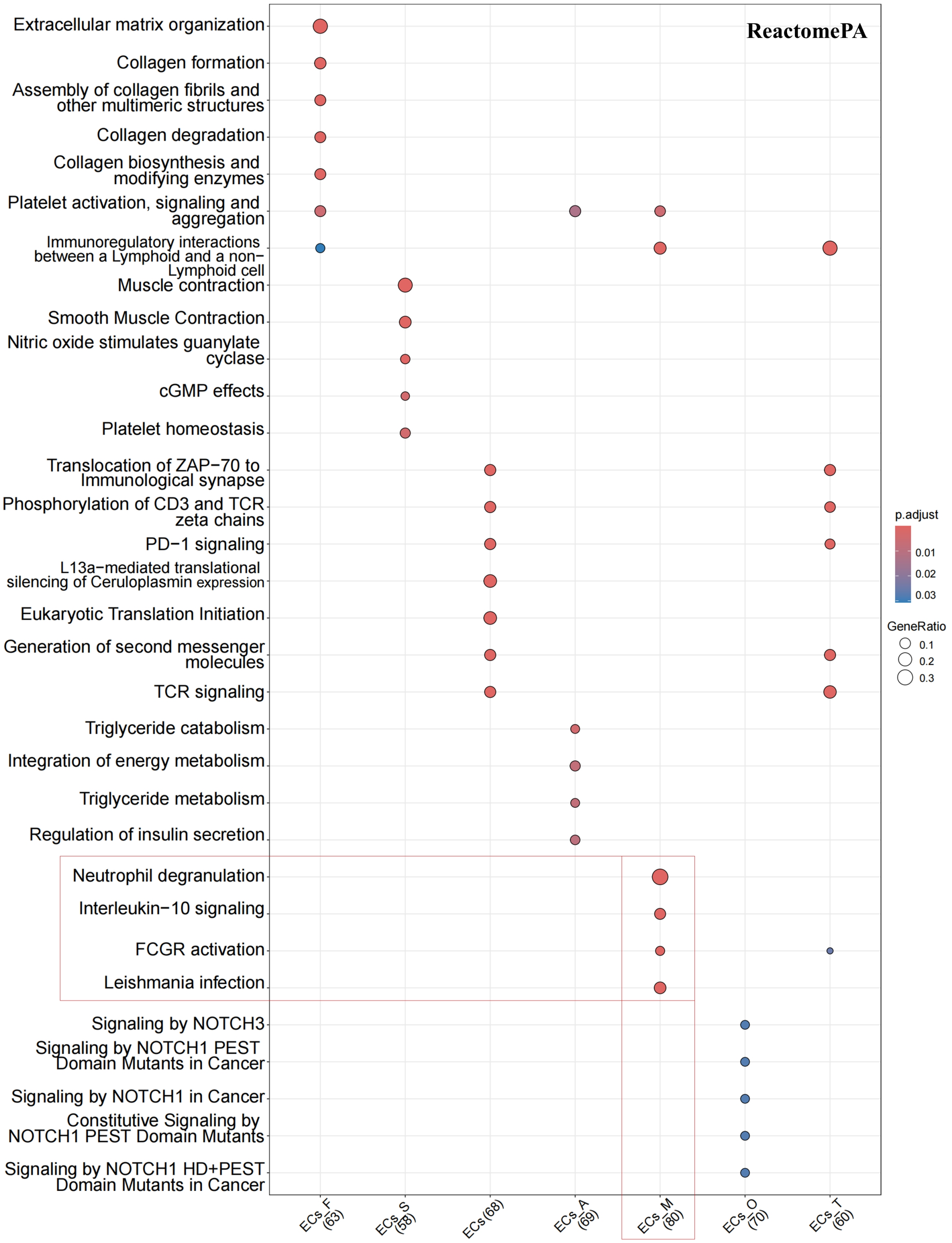

Supplementary Fig. 14. Reactome pathway enrichment analysis of endothelial cell (EC) subpopulations in human thoracic aortic dissection (TAD) tissue. Reactome pathway enrichment analysis was performed using differentially expressed genes from EC subpopulations identified in TAD tissue. Each dot represents a significantly enriched pathway in the corresponding EC subpopulation. Dot size indicates the gene ratio (GeneRatio), and color represents the adjusted p

value (p.adjust). The boxed area highlights pathways enriched in the EC_M subpopulation, including neutrophil degranulation, interleukin-10 signaling, and FCGR activation.

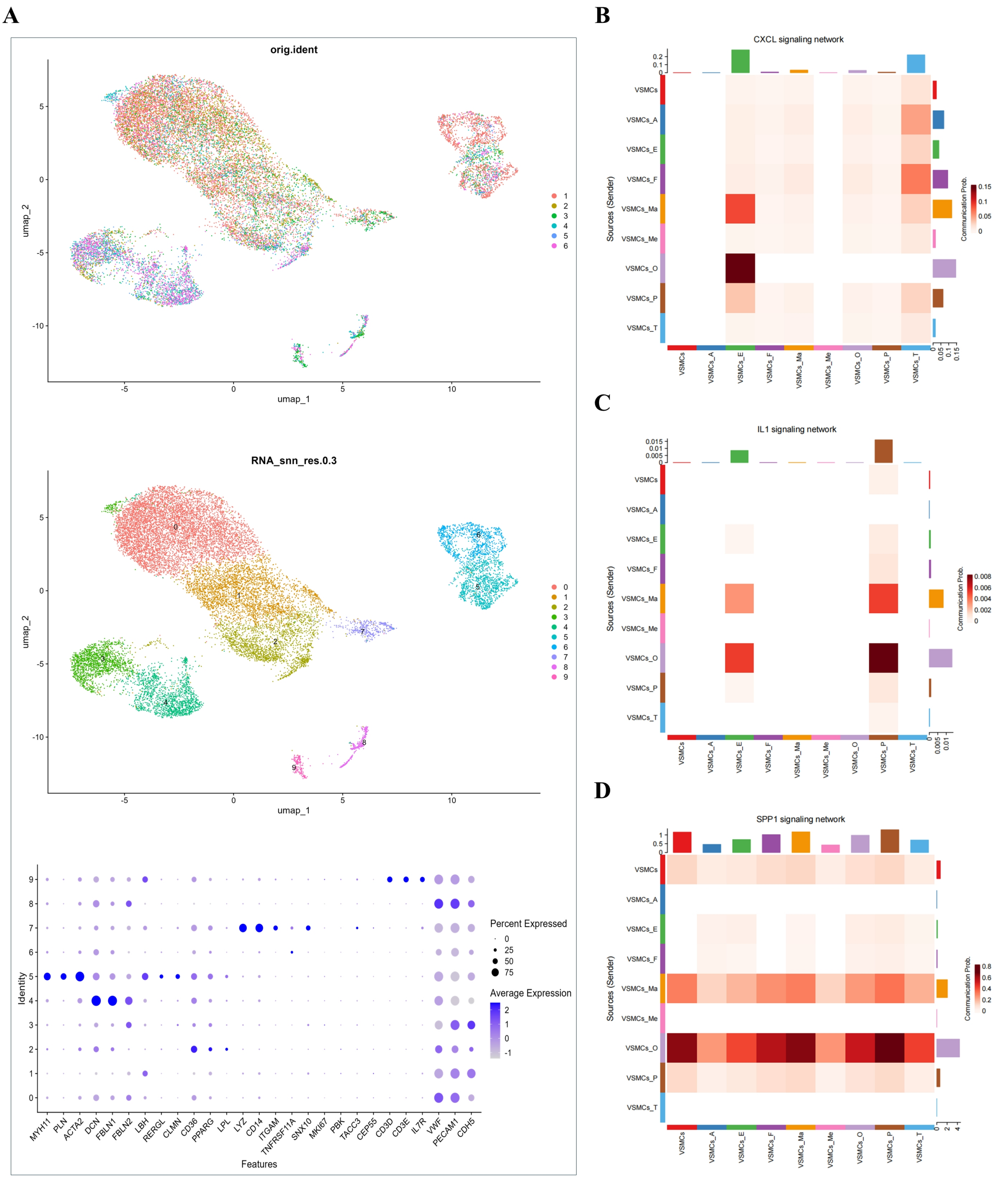

Supplementary Fig. 15. Vascular smooth muscle cell (VSMC) subpopulation clustering and intercellular communication analysis in human thoracic aortic dissection (TAD) tissue. (A) UMAP visualization of VSMC subpopulations identified in TAD tissue. The upper panel shows cells colored by sample identity (orig.ident), and the middle panel shows unsupervised clustering results (resolution = 0.3). The lower panel presents a dot plot of representative marker genes used for VSMC subpopulation annotation. **(B)** Predicted CXCL signaling network among VSMC subpopulations inferred using the CellChat algorithm. **(C)** Predicted IL-1 signaling network among

VSMC subpopulations inferred using CellChat. **(D)** Predicted SPP1 signaling network among VSMC subpopulations inferred using CellChat. Heatmaps indicate the communication probability between sender and receiver VSMC subpopulations, and the relative signaling roles of each subpopulation are summarized above the heatmaps.

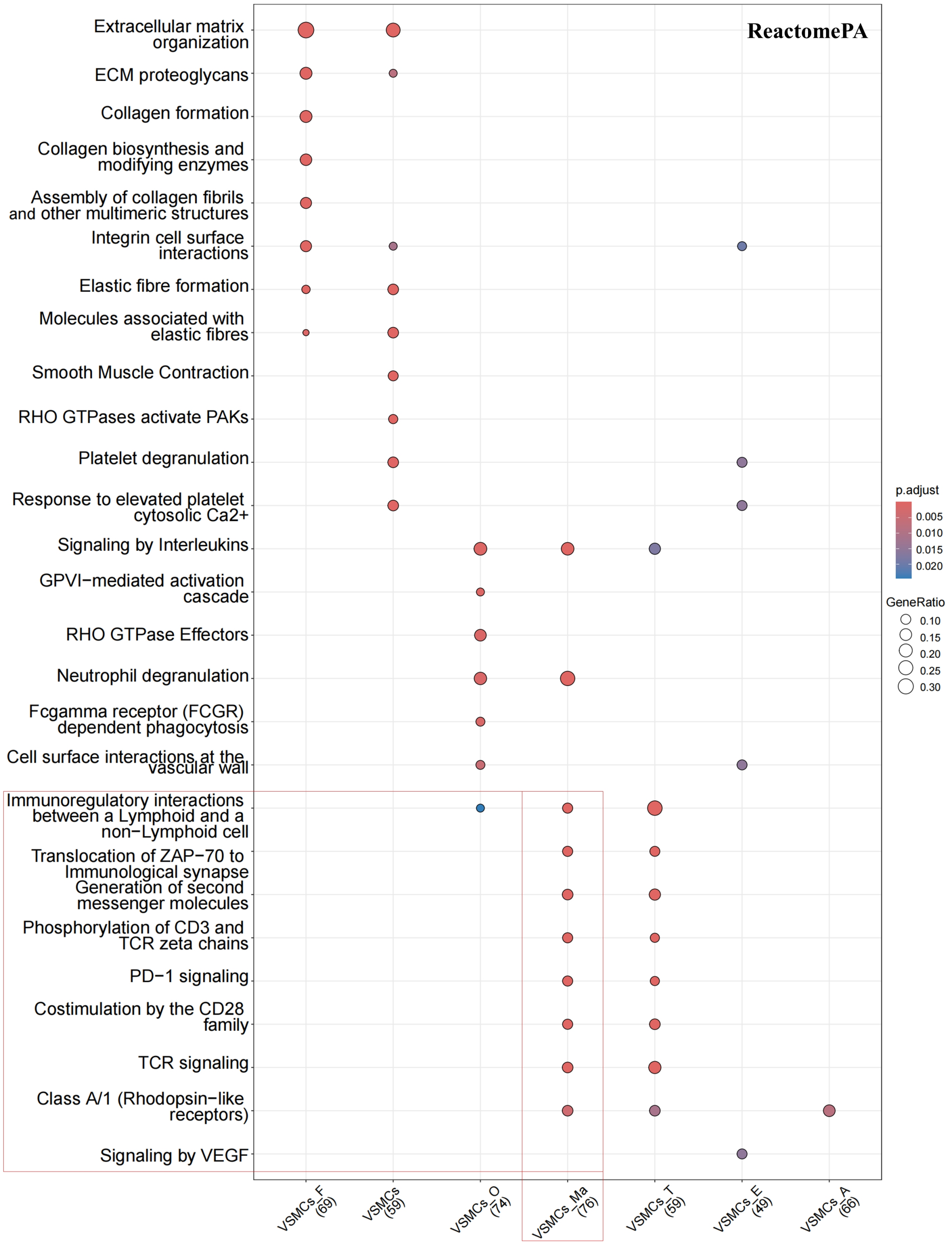

Supplementary Fig. 16. Reactome pathway enrichment analysis of vascular smooth muscle cell (VSMC) subpopulations in human thoracic aortic dissection (TAD) tissue. Reactome pathway enrichment analysis was performed using differentially expressed genes from VSMC subpopulations identified in TAD tissue. Each dot represents a significantly enriched pathway in the corresponding VSMC subpopulation. Dot size indicates the gene ratio (GeneRatio), and color

represents the adjusted p value (p.adjust). The boxed region highlights immune-related pathways enriched in the VSMC_Ma subpopulation, including T cell receptor (TCR) signaling, PD-1 signaling, and CD28 costimulation.
